# On the possible use of hydraulic force to assist with building the step pyramid of saqqara

**DOI:** 10.1371/journal.pone.0306690

**Published:** 2024-08-05

**Authors:** Xavier Landreau, Guillaume Piton, Guillaume Morin, Pascal Bartout, Laurent Touchart, Christophe Giraud, Jean-Claude Barre, Cyrielle Guerin, Alexis Alibert, Charly Lallemand

**Affiliations:** 1 Paleotechnic., Paris, France; 2 Univ. Grenoble Alpes, INRAE, CNRS, IRD, Grenoble INP, IGE, Grenoble, France; 3 Sicame Group, Arnac-Pompadour, France; 4 CEDETE—Centre d’études sur le Développement des Territoires et l’Environnement, Université d’Orléans, Orléans, France; 5 AtoutsCarto, Bourges, France; 6 Verilux International, Brienon-sur-Armançon, France; Israel Antiquities Authority, ISRAEL

## Abstract

The Step Pyramid of Djoser in Saqqara, Egypt, is considered the oldest of the seven monumental pyramids built about 4,500 years ago. From transdisciplinary analysis, it was discovered that a hydraulic lift may have been used to build the pyramid. Based on our mapping of the nearby watersheds, we show that one of the unexplained massive Saqqara structures, the Gisr el-Mudir enclosure, has the features of a check dam with the intent to trap sediment and water. The topography beyond the dam suggests a possible ephemeral lake west of the Djoser complex and water flow inside the ’Dry Moat’ surrounding it. In the southern section of the moat, we show that the monumental linear rock-cut structure consisting of successive, deep compartments combines the technical requirements of a water treatment facility: a settling basin, a retention basin, and a purification system. Together, the Gisr el-Mudir and the Dry Moat’s inner south section work as a unified hydraulic system that improves water quality and regulates flow for practical purposes and human needs. Finally, we identified that the Step Pyramid’s internal architecture is consistent with a hydraulic elevation mechanism never reported before. The ancient architects may have raised the stones from the pyramid centre in a volcano fashion using the sediment-free water from the Dry Moat’s south section. Ancient Egyptians are famous for their pioneering and mastery of hydraulics through canals for irrigation purposes and barges to transport huge stones. This work opens a new line of research: the use of hydraulic force to erect the massive structures built by Pharaohs.

## 1 Introduction

The funerary complex of King Djoser, built at Saqqara in Egypt around 2680 B.C., is considered a significant milestone in monumental architecture. It is the first to disclose two crucial innovations: a pyramid shape for the pharaoh’s grave and the exclusive use of fully dressed stones for masonry. In practice, it is also revolutionary in the ability to extract and raise stones by millions before stacking them with precision [1]. Djoser’s complex visible achievements are such that its architect, Vizier, and Great Priest of Ra, Imhotep, was deified by the New Kingdom.

The knowledge and innovations implemented in the Djoser mortuary complex profoundly influenced future developments and were widely perfected throughout the Old Kingdom’s III^rd^ and IV^th^ Dynasties, *i*.*e*. circa 2680–2460 B.C. These developments resulted in a substantial increase in the megaliths’ size [2], leading to pyramids of spectacular dimensions, such as those of the Meidum, Dahshur, and Giza plateaus. In less than 150 years, the average weight of the typical large stones was thus multiplied by ≈8 and went from ≈300 kg for Djoser’s pyramid to more than 2.5 tons for Chephren’s pyramid’s structural blocks [3]. For the largest lintels, the weight increases by two orders of magnitude, with several blocks of ≈50 – 100 tons for Cheops’ pyramid. On this short timeframe on the scale of human history, Egyptians carried and raised some 25 million tons of stones [4] to build seven monumental pyramids. Assuming an annual work schedule of 300 days at a rate of 10 hours/day, meaning 450,000 hours spread over less than 150 years, this requires a technical and logistical organization capable, on average, of cutting, moving, and adjusting about 50 tons of stone blocks per hour. Even if one admits that not every pyramid’s blocks are fitted with millimeter precision, the amount of work accomplished is truly remarkable. Interestingly, the pyramids later built in Egypt tended to be smaller with time and never reached the volume of the Old Kingdom’s monumental structures again.

As authentic sources from the working sphere of pyramid architects are currently lacking, no generally accepted wholistic model for pyramid construction exists yet. Although many detailed publications dedicated to pyramid-building procedures have given tangible elements [5, 6], they usually explain more recent, better documented, but also smaller pyramids [7]. These techniques could include ramps, cranes, winches, toggle lifts, hoists, pivots, or a combination of them [8–10]. Studies of the pyramid’s construction sites also revealed a high level of expertise in managing the hydraulic and hydrological environment, such as utilizing waterways to deliver materials, constructing ports and locks, or setting up irrigation systems [11, 12]. These achievements have led some scholars to refer to ancient Egypt as an ‘early hydraulic civilization [11].’ However, there is actually very little multidisciplinary analysis combining the rich archaeological findings on pyramids with other disciplines such as hydrology, hydraulics, geotechnics, paleoclimatology, or civil engineering [9]. Therefore, the topic of water force in the context of pyramid construction remains insufficiently addressed in the academic literature.

Moreover, a second question accentuates the enigma: the Pharaohs who built these pyramids are missing. Until now, neither written record nor physical evidence reports the discovery of one of the III^rd^ and IV^th^ Dynasties’ Pharaohs. Old Kingdom’s ‘big’ pyramids’ rooms were allegedly plundered [13–15] during the millennia that followed the construction of the pyramids, leaving little evidence behind [12]. The III^rd^ and IV^th^ Dynasties’ rooms present little or no funerary attributes, such as those observed in other high-dignitary figures’ tombs contemporary to the period [16, 17], with no King’s remains found inside. In addition, the walls of the pyramids’ chambers do not exhibit any hieroglyphs, paintings, engravings, or drawings, which would allow us to qualify them as funerary with certainty. Despite this lack of evidence, many authors [18] still support that these rooms can be attributed to Pharaohs’ burials mainly based on royal cartouches or Kings’ names found elsewhere within the pyramid or nearby temples.

Over the recent years, Dormion & Verd’Hurt [19, 20], Hamilton [21–24] or others [1, 25] were among the first to consider possible non-funerary functions of pyramids’ internal layouts by pointing out some architectural inconsistencies and highlighting the high degree of complexity of several structures, irrelevant for a burial chamber. Their analysis provided both chambers and gallery systems with a technical dimension, emphasizing a level of engineering on the part of the ancient builders that is quite remarkable and sometimes challenges any apparent explanation. This technical level is at once reflected in the geometry of the rooms and ducts, as well as in the stonework, which includes materials selection, extraction, cutting, and then assembling with exceptional accuracy [20]. This precision involved several advanced sub-techniques, such as inter-block mortar joint realization [26–29] or stone polishing with flatness and roughness values that reach levels of contemporary know-how. Apart from surfaces and interfaces, the builders’ technical ability is also evident throughout sophisticated mechanical systems set up in the pyramids [30], as swivel stone flaps’ designs in the Meidum and Bent pyramids [21, 24] or tilted portcullises found in the Bent pyramid, as well as at Giza [20]. These elements suggest that, rather than an aesthetic rendering or a funerary use for these layouts, ancient Egyptians intended technical functions for some walls, tunnels, corridors, shafts, and chambers where more straightforward existing techniques were insufficient.

In summary, the analysis of the pyramids’ construction and the investigation of their internal layouts seem to require more research to provide a wholistic explanation to their purpose. This study aims to provide a fresh look at these topics by applying an alternative, multi-disciplinary, wholistic approach. It revisits the Old Kingdom’s pyramids’ construction methodology and seeks to explain the significance of internal layouts during construction. Based on current archaeological knowledge, we demonstrate that the Saqqara’s topography and the layout of several structures are consistent with the hypothesis that a hydraulic system was used to build the pyramid. The paper is divided into three main sections that analyze the current scientific literature to address the following inquiries: (i) Was the plateau of Saqqara supplied with water? (ii) If so, how was it possibly stored and treated? and (iii) How was it used to build the pyramid? A discussion and some concluding remarks and perspectives follow.

## 2 The saqqara’s hydrologic network

Our study began with the postulate that the larger Cheops’ and Chephren’s pyramids of Giza plateau were the outcomes of technical progress from previous pyramids, with the Step Pyramid as a technological precursor. While many literature studies focus on the construction of Cheops’ pyramid, we found it more relevant to examine the building techniques used for the Step Pyramid first. This would provide insight into the processes used by ancient builders that were later refined in subsequent pyramids. As a first approach, we analyzed potential reasons for the specific building of King Djoser’s Complex on the Saqqara Plateau.

### 2.1 Water resource from the desert wadis

Although detailed measurements of the Nile flood levels have been reported since the V^th^ Dynasty (2480 B.C.) [31–33], there is very little information available about the hydrology of its desert tributaries, known as ’wadis’, in ancient Egypt. Sedimentological evidence of heavy rainfalls and flash floods exists [31, 34] but little is known beyond that.

Determining the rainfall regime that the Saqqara region experienced about 4,700 years B.P. is challenging and uncertain. Past studies demonstrated that, from about 11,000 to 5,000 B.P, during the so-called ‘Green Sahara’ period, the whole Sahara was much wetter than today, and the landscape was savannah rather than desert [35, 36]. Around 4500–4800 years B.P. too, the Eastern Mediterranean region was wetter than it is now, despite drying up later [37–39]. A range of annual precipitation value of 50–150 mm/year is assumed in the following calculation to perform crude computations of water resource. It covers the range between the >150 mm/year suggested by Kuper & Kropelin [40] for the end of the Green Sahara period, before the subsequent drier period, during which rainfall decreased to <50 mm/year. The range of variability, *i*.*e*. 50 to 150 mm/yr is also consistent with the typical inter-annual rainfall variability observed in the region [38].

Then, current hydrological monitoring on Egyptian wadis located further to the north and experiencing comparable annual rainfall (*i*.*e*., 100–200 mm/yr) showed that only 1–3% of this mean annual precipitation was measured as runoff, i.e., surface flows [41]. This average range is hereafter used for conservative, first-order estimations of available water volume, referred hereafter to as the ‘water resource’. Note that the infrequent, most intense events can reach 50 mm of rainfall and trigger devastating flash floods where the runoff coefficients have been measured up to 30%, *i*.*e*., one order of magnitude higher than the mean annual [41–43]. Note that these water resource and flash flood hydrology estimates neglect that the soils were probably richer in clay and silt just after the Green Sahara period, with several millennia of a wetter climate and savannah landscape [35, 36], which would increase the runoff coefficient and available surface water resource in the wadis.

### 2.2 The Saqqara site: a plateau with a water supply

**Fig 1:**
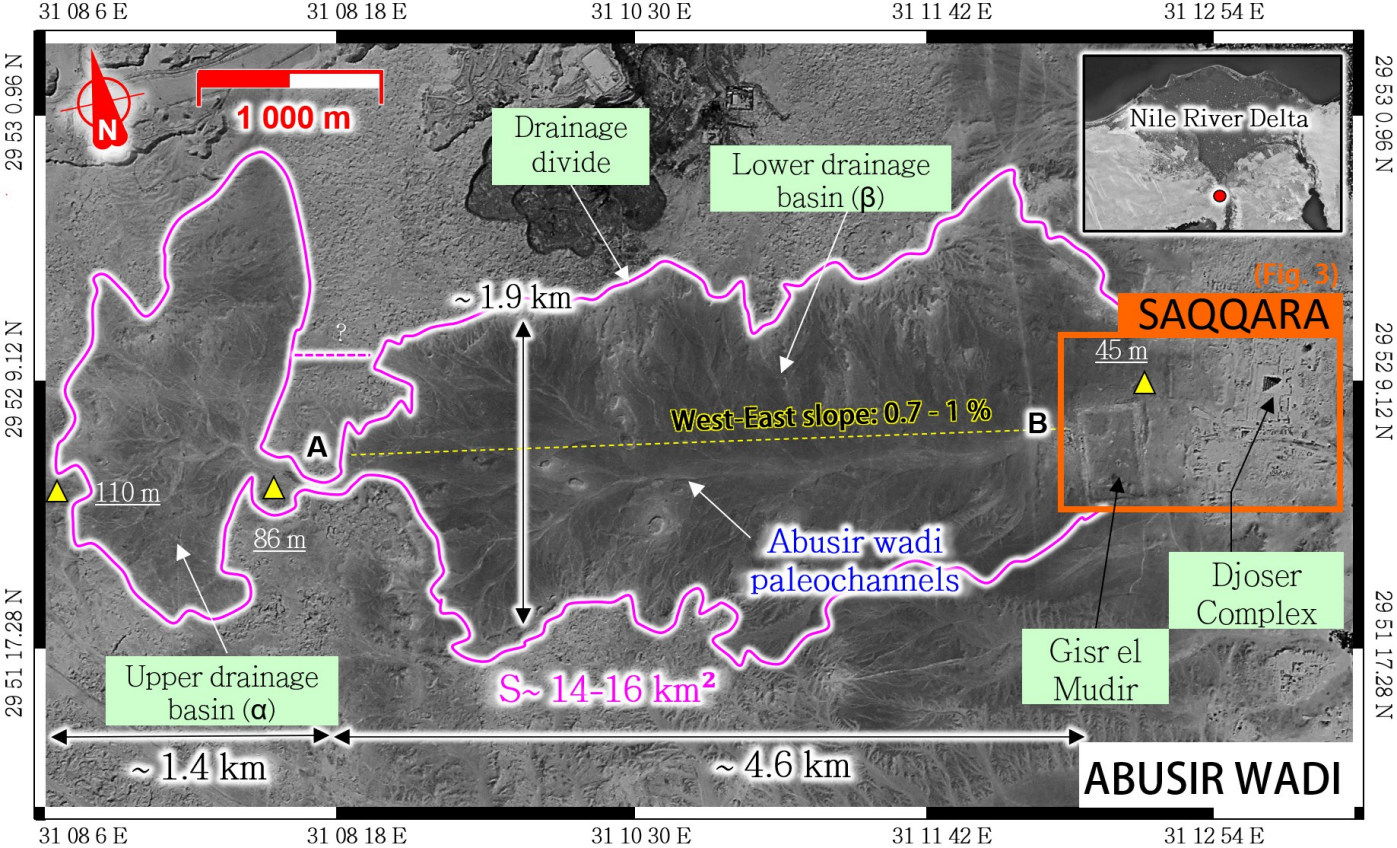
General location of the Djoser complex at Saqqara (inset) and drainage area of the Abusir wadi, west of the Gisr el-Mudir enclosure. (Satellite image: Airbus Pléiades, 2021-07-02, reprinted from Airbus D&S SAS library under a CC BY license, with permission from Michael Chemouny, original copyright 2021).

The Saqqara necropolis is located on a limestone plateau on the west bank of the Nile River, about 180 km from the Mediterranean Sea (**Fig 1**). The entire site lies in the desert, less than two kilometers from the plateau’s edge (elevation 40–55 m ASL—*Above Sea Level*), which overlooks the Nile floodplain (height ≈ 20 m ASL). Further to the west, the desert rises gently for about 20 km (hills’ top elevation ≈ 200–300 m ASL).

The reasons behind the construction of the Djoser complex at Saqqara remain unclear. The contribution of economic, socio-political, and religious factors was previously highlighted [44, 45], but environmental factors were also possibly influential. In 2020, Wong provided evidence that the climate, geology, and hydrology would have influenced building choices and may have contributed to, or perhaps accelerated, the emergence of stone architecture on the Saqqara plateau [37].

From a geological standpoint, the layered structure of the limestone at Saqqara was indeed stressed as a favorable factor for excavating large amounts of construction stones [46, 47]. These layers, which consist of 30–60 cm thick sand-rich calcareous beds alternated with calcareous clay and marl layers, made it easy to extract the limestone blocks from their parent beds by vertical cuttings, the original thickness being reflected in the building stones’ thickness of Djoser’s complex.

From a hydrological standpoint, the Abusir wadi is considered a second environmental factor that strongly influenced the Early Dynastic development of the Saqqara necropolis at least [45, 48–50]. The Abusir wadi is the ephemeral stream draining the hills west of Saqqara (**Fig 1)**. Before this study, academic research mainly focused on the downstream part of the wadi [45, 48–50], namely the Abusir Lake [51] located north of Saqqara Plateau. However, the upstream portion has remained undocumented.

In order to analyze the relationships between the Abusir wadi and the Step Pyramid’s construction project, the drainage networks west of the Saqqara area were mapped for the first time to the best of our knowledge, using various satellite imagery (**Fig 1**) and Digital Elevation Models (see **S1 Fig** in S2 File).

A paleo-drainage system can be identified upstream of the Gisr el-Mudir structure as the origin of the Abusir wadi (**Fig 1**, pink line). The boundaries of this runoff system form a catchment area never reported so far, although easily recognizable from the geomorphological imprints of surface paleochannels in the desert and on historical maps [52]. Although it currently has a 15 km^2^ surface area, we cannot rule out the possibility that the drainage divides shifted and changed due to land alterations and aeolian sand deposits over the past 4,500 years.

The current catchment summit is about 110 m ASL, giving the Abusir wadi a 1% average slope over its slightly more than 6 km length. In the field of hydrology, a 1% gradient is described as ‘rather steep’. With such steep slopes, transportation of sand and gravel is expected during flashfloods, which can cause severe downstream damage (scouring or burying of structures, filling of excavations and ponding areas). In comparison, irrigation channels are rather at least ten times less steep (about 0.1%), and the Nile slope is less than 0.01% (less than 200m of elevation gain between Aswan and Cairo).

### 2.3 The Wadi Taflah: A possible complementary water supply

**Fig 2:**
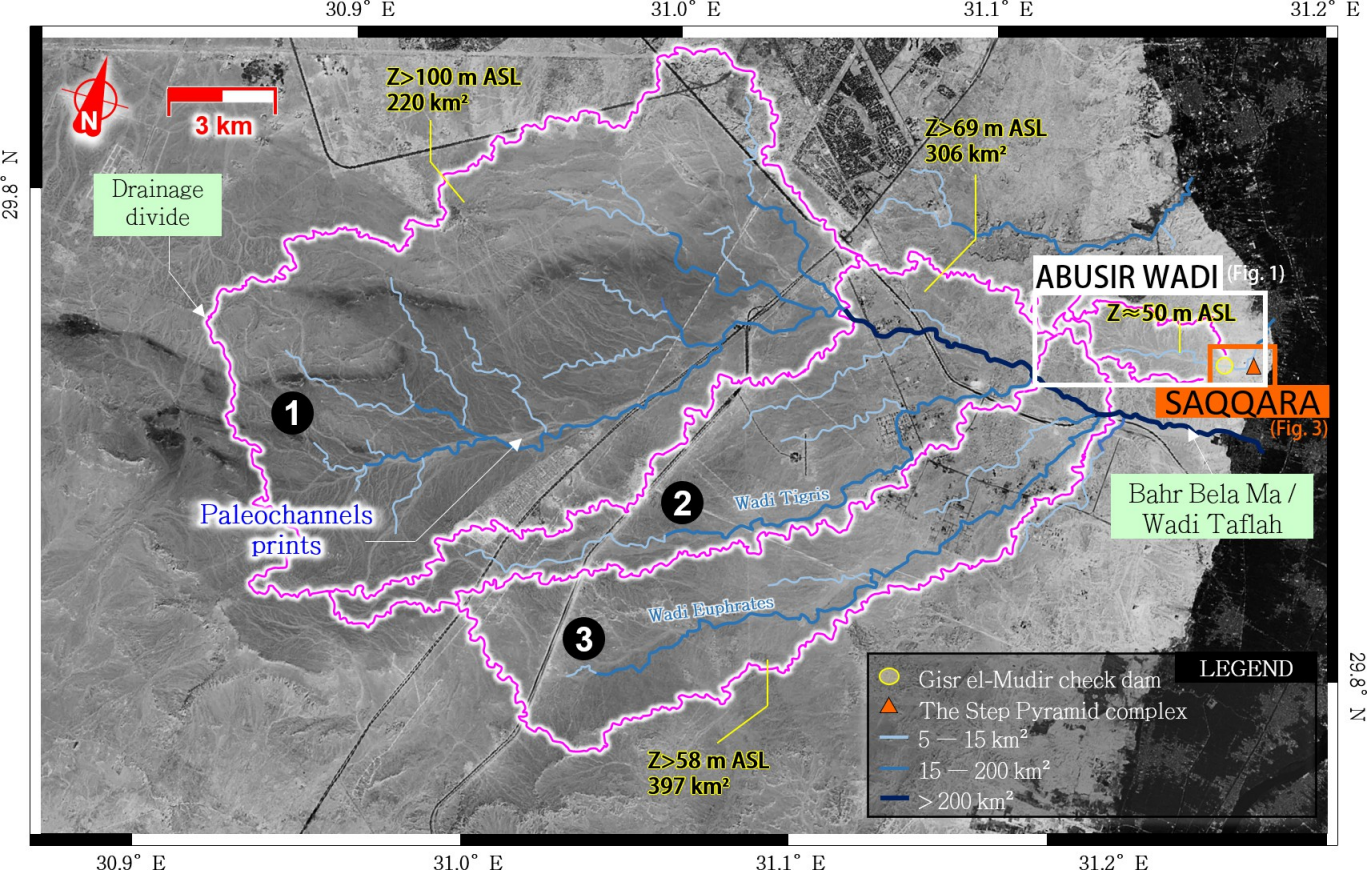
Drainage area of the Wadi Taflah, about 2 km south of the Djoser Complex. (Satellite image: Airbus Pléiades, 2021-07-02, reprinted from Airbus D&S SAS library under a CC BY license, with permission from Michael Chemouny, original copyright 2021).

Reported since the early 1800s, a former tributary to the Nile called the *Bahr Bela Ma* [53, 54] or ‘*Wadi Taflah’* flowed parallel to the Abusir wadi catchment, less than two kilometers south of the Saqqara plateau. From satellite imagery, we identified that the Wadi Taflah arises from a drainage area of almost 400 km^2^ and consists of three main branches (**Fig 2**, numbered black dots) still visible from the desert’s geomorphological marks. This network is also visible on the radar imagery provided by Paillou [55] that can penetrate multiple meters of sand (**S2 Fig** in S2 File). The similarity of the optical and radar drainage patterns confirms the existence and old age of this hydrological network.

Although no canal was detected from the satellite data, the close proximity of Abusir wadi with the Wadi Taflah (**Fig 2**) is intriguing and raises the question about a potential ancient, artificial connection between them. According to the 18^th^-century maps published by Savary [54], the Wadi Taflah was ‘closed by an ancient King of Egypt.’ Such a testimony, although imprecise, could suggest the construction of a water diversion by a former ruler. A geophysical investigation could help to find such a structure if existing. The drainage area of Wadi Taflah covers nearly 400 km^2^ at an elevation >58 m ASL. This elevation is high enough to allow the diversion of the drainage area toward the Abusir wadi. This would result in an increase in the drained area and associated availability of water resources by a factor of >25 times. Based on the hydrological conditions described in section 2.1, the estimated water resource from Abusir wadi and Wadi Taflah is crudely between 7,500 to 68,000 m^3^/year and 200,000 to 1,800,000 m^3^/year, respectively.

### 2.4 The Abusir wadi: A structural element in the early dynastic Saqqara’s development

**Fig 3:**
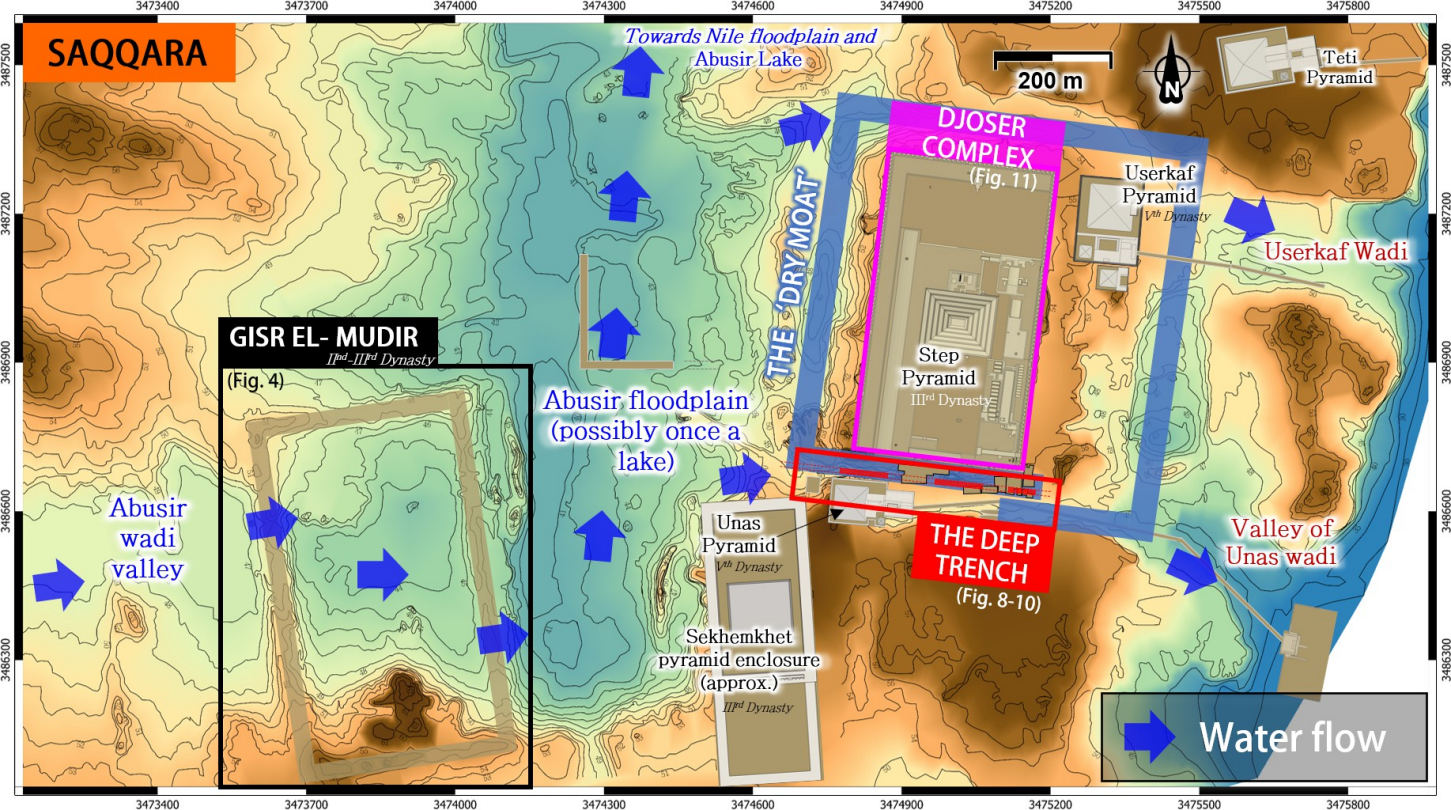
Topography of north Saqqara. Contour lines extracted from the 1:5,000 topographical map [52] “Le Caire, sheet H22”.

**Fig 4:**
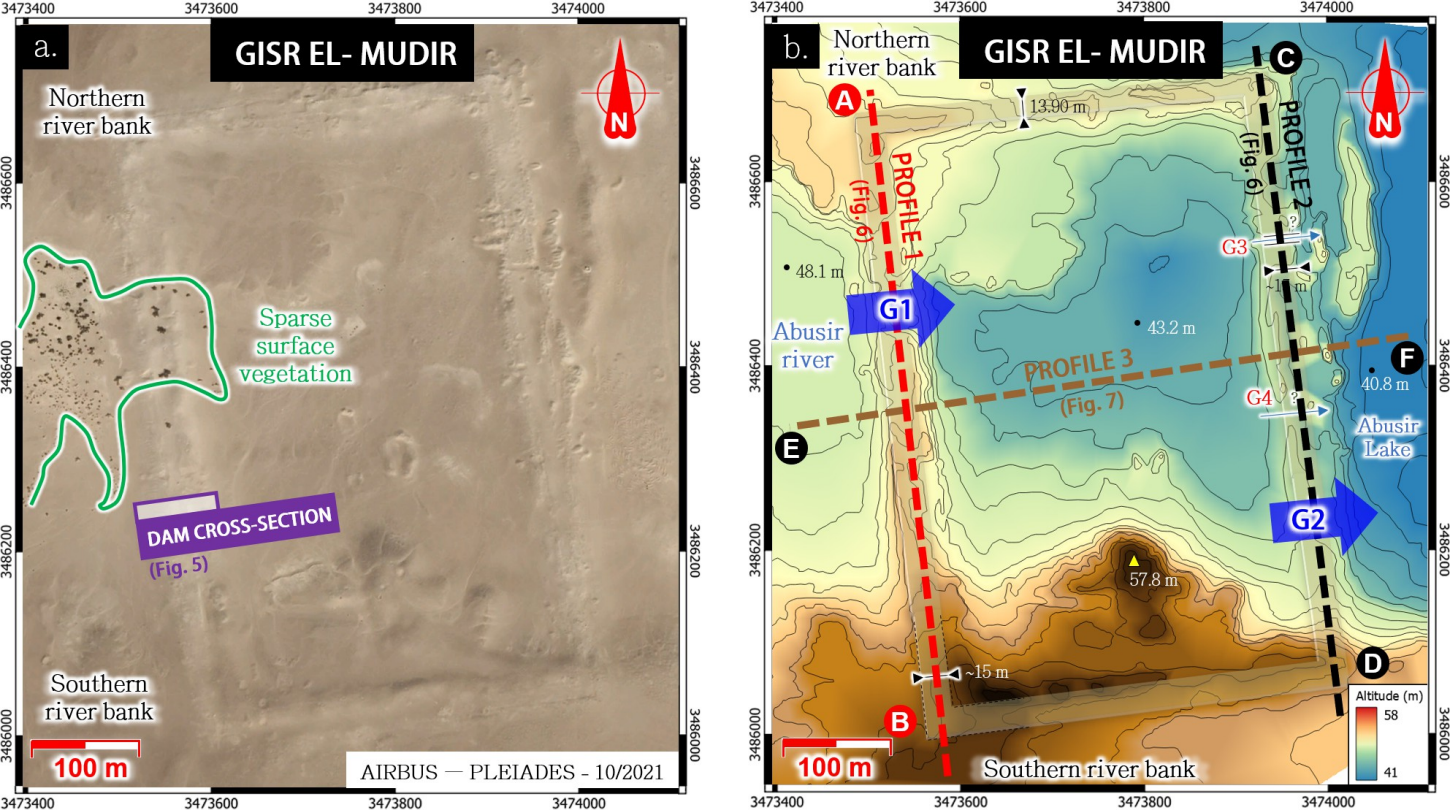
a. The Gisr el-Mudir check dam (Satellite image: Airbus Pléiades, 2021-07-02, reprinted from Airbus D&S SAS library under a CC BY license, with permission from Michael Chemouny, original copyright 2021); b.: Digital Elevation Model generated from the 1:5,000 topographical map “Le Caire, sheet H22”.

According to the Saqqara topography (**Fig 3**), the Abusir wadi flowed through the Gisr el-Mudir enclosure before heading north towards the Nile floodplain, where it used to feed an oxbow lake, the Abusir Lake [51]. With such a localization, the Gisr el-Mudir walls literally dam the Abusir wadi valley’s entire width. The sparse vegetation only growing in the valley bottom upstream of Gisr el-Mudir and not elsewhere in the area evidences this damming and interception of surface and subsurface flows (**Fig 4A,** green line). This slight moist area is dominated by plants commonly found in desert margins and wadis, such as *Panicum thurgidum* and *Alhagi graecorum [56],* and is typical of hypodermal flows.

Downstream of the Gisr el-Mudir, the Abusir wadi joins the Saqqara Plateau. Its boundaries are defined to the south by an outcropping limestone ridge and to the east by the Sekhemkhet and Djoser’s enclosures (**Fig 3**).

The landform of this area seems inconsistent with a pure fluvial formation. Instead, the very flat topography on about 2–2.5 km^2^_,_ according to the *Saqqara Geophysics Survey Project* (SGSP) [57–60] and possibly allowed some ephemeral ponding water which may have resulted in an episodic *upper* Abusir lake after the most intense rainfalls. However, due to the several-meter deep wind-blown and alluvial sand cover accumulated over the past millennia [57], the riverbed altitudes during Djoser’s reign are challenging to establish without further investigations, and only broad patterns can be determined from the local topography [52].

As with many other small wadis, the Early Dynastic hydrology of the Abusir wadi remains largely unknown. According to fluvial sediment analysis in the Abusir Lake area, the Abusir wadi was probably a perennial stream during the Old Kingdom period [51]. Although the climate is hot and arid nowadays, several studies support a more humid environment during the Old Kingdom *[34]*. Multiple strands of evidence indeed suggest that Egypt experienced considerable rainfalls around the reign of Djoser, resulting in frequent flooding and heavy runoffs on the Saqqara Plateau. This climatic feature is supported by sedimentary deposits resulting from flowing water of ‘considerable kinetic force’ contemporary to Djoser’s reign *[61, 62]*. According to Trzciński et al.[34], the strongly cemented structure L3 found in the Great Trench surrounding the Djoser Complex was due to cyclical watering while the high content of Fe3+ indicates that the region experienced intensive weathering in a warm and humid environment. In 2020, Wong concluded that the ‘*intriguing possibility that the Great Trench that surrounds the Djoser complex may have been filled with water*’ during Djoser’s reign [37]. If so, this might explain why tombs were built on the northern part of the Saqqara plateau which has a higher altitude *[45]* and nothing was constructed inside the Trench until the reign of Userkaf and Unas (V^th^ Dynasty).

## 3. The saqqara’s water management system

### 3.1 The Gisr el-Mudir check dam

Reported at least since the 18^th^ century [63] and extensively described within a decade of a geophysical survey by Mathieson *et al*., see also [45] for a summary, the Gisr el-Mudir is a rectangular enclosure located a few hundred meters west of the Djoser’s complex (**Fig 3**, **Fig 4A & 4B**). This monumental structure has a footprint of about 360 m x 620 m, *i*.*e*., larger than the Djoser complex (545 m x 277 m). The walls have an estimated volume of >100,000 m^3^ (SGSP, 1992–1993 report), meaning about one-third of the Step Pyramid’s volume. Field inspection and geophysical results from the SGSP [57] found no construction inside except for a couple of more recent, small graves, thus confirming that the enclosure is mainly empty. Moreover, several elements in the building suggest that this structure predated the Step Pyramid’s complex and was tentatively dated to the late II^nd^ or early III^rd^ Dynasty [57, 64], which might turn it into the oldest substantial stone structure in Egypt discovered so far.

Before this study, several conflicting theories about the Gisr el-Mudir’s purpose were put forward [59]: *e*.*g*., an unfinished pyramid complex (but the lack of a central structure made it improbable to be a funerary monument), a guarded fortress [65] protecting the Saqqara necropolis from nomadic Bedouin incursions, an embankment to raise a monument to a higher level [66], a celebration arena [64, 67], or even a cattle enclosure. However, given the low level of exploratory work afforded to the structure, no generally accepted explanation exists yet, and its purpose has remained more conjectural than substantiated.

**Fig 5:**
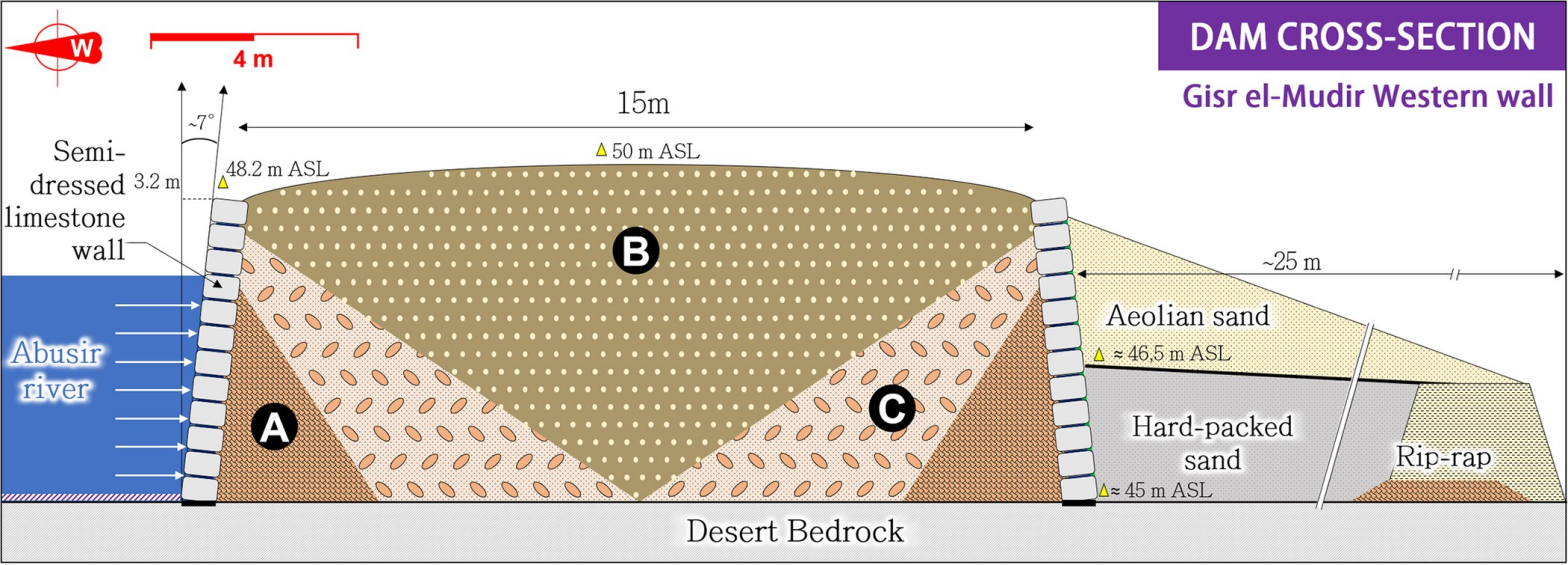
Cross-section of the Gisr el-Mudir’s west wall (location on Fig 4A., purple rectangle). Figure adapted from [58].

In light of the upstream watershed and its transversal position across the Abusir River, the Gisr el-Mudir’s western wall meets the essential criteria of a check dam, *i*.*e*., a dam intending to manage sediment and water fluxes [68, 69]. This comparison is particularly striking regarding its cross-section (**Fig 5**). According to Mathieson *et al*. [59], the basic structure of this wall consists of a hollow construction of two rough-hewn limestone masonry skin-walls, ≈3.2 m high, separated by a 15 m interspace filled with three layers of materials extracted from the surrounding desert bedrock [70] and cunningly arranged. The first layer (**Fig 5**, ‘**A**’ dot) is made of roughly laid local limestone blocks forming a buttress against the inside of the facing blocks. The secondary fill (**B**) comprises coarse sand and medium to large limestone fragments. Then, the third fill (**C**) consists of rough to fine sand and silt, small limestone fragments, and chippings with pebble and flint nodules. Finally, these A, B, and C backfill layers are positioned symmetrically to the median axis of the wall.

Civil engineering was used during the Old Kingdom to protect settlements from flash floods, such as the Heit el-Ghurab (’Wall of the Crow’) safeguarding the village of the pyramid builders at Giza [71]. Regarding the Gisr el-Mudir structure, the abovementioned elements strikingly echo the transversal profile and slope protection of another famous Old Kingdom structure: the Sadd el-Kafara dam built on the Wadi al-Garawi, a colossal building found to be contemporary to that of the Gisr el-Mudir [72–74]. Both structures present the technical signature of zoned earthen dams: a wide embankment made of a central impervious core surrounded by transition filters, *i*.*e*., filling material with coarser grain size, preventing erosion, migration, and potential piping of the core fine material due to seepage. The semi-dressed limestone walls stabilized the inner material and protected it against erosion when water flowed against and above the dam. Both dams have much broader profiles than modern dams. This oversizing could be due to the unavailability of contemporary compaction systems or an empirical and conservative structural design. They both have narrower cores of fine material at the bottom of the dam than at their crest, contradicting modern design [75]. This can be attributed to the construction phasing that would have started by raising the sidewalls buttressed against the coarse and intermediate filling (**B** and **C** fills in **Fig 5**), followed by a phase of filling the wide core with finer, compacted material [72].

**Fig 6:**
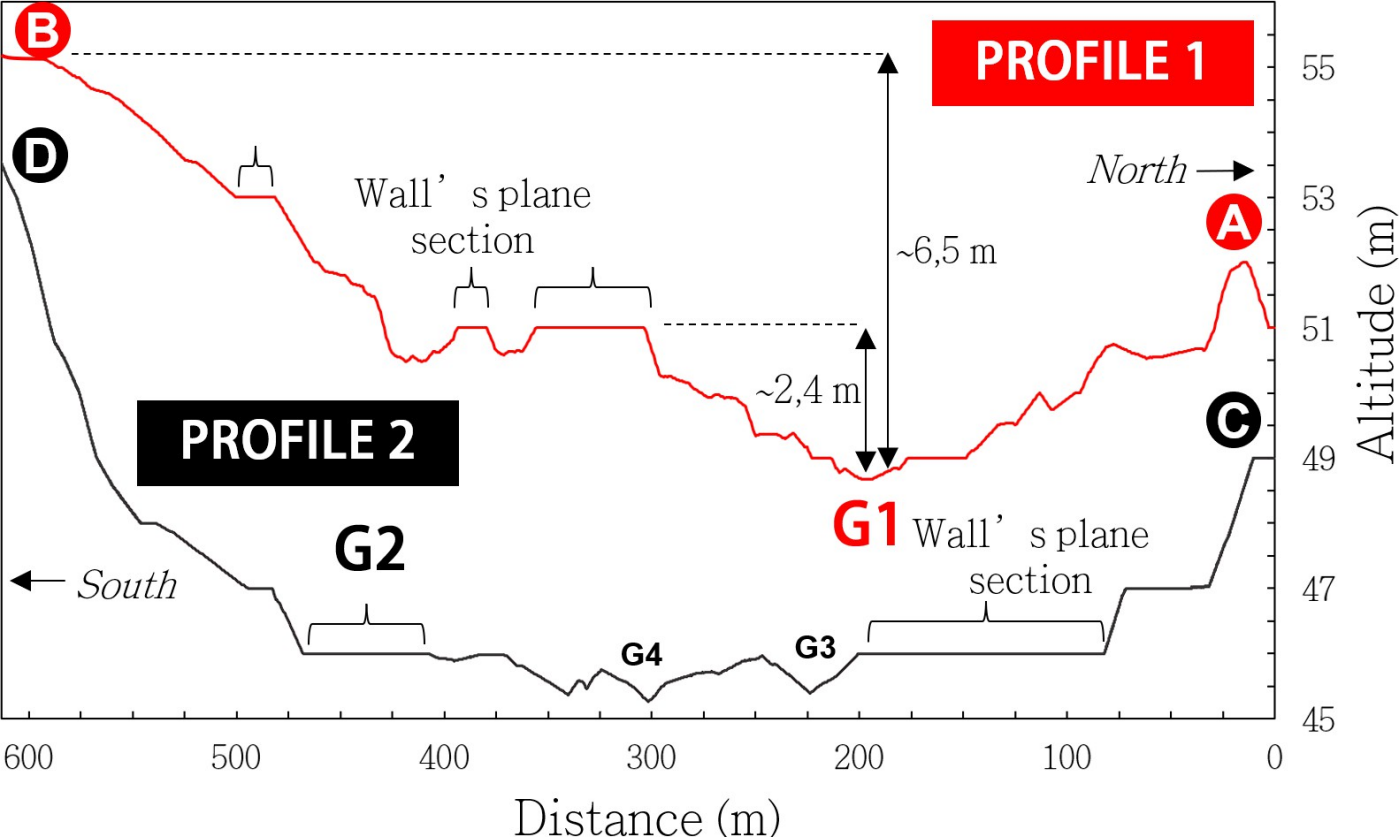
Elevation profiles along the eastern (red line) and western (black line) walls.

Finally, the eastern wall’s north-south profile (**Fig 6**, line **A-B**) presents a parabolic profile relevant to guide the flows to the basin’s center formed by Gisr el-Mudir. This guidance would have prevented the dam failure by outflanking during flooding events when the dam outlet was saturated. We estimate that the accumulated water crossed the dam through an outlet likely located at the valley’s lowest elevation, *i*.*e*., near 48.7 m ASL (**G1** in **Figs 4B** and **Fig 6**). In summary, the Gisr el-Mudir’s western wall likely acted as a first check dam to the Abusir wadi flows.

**Fig 7:**
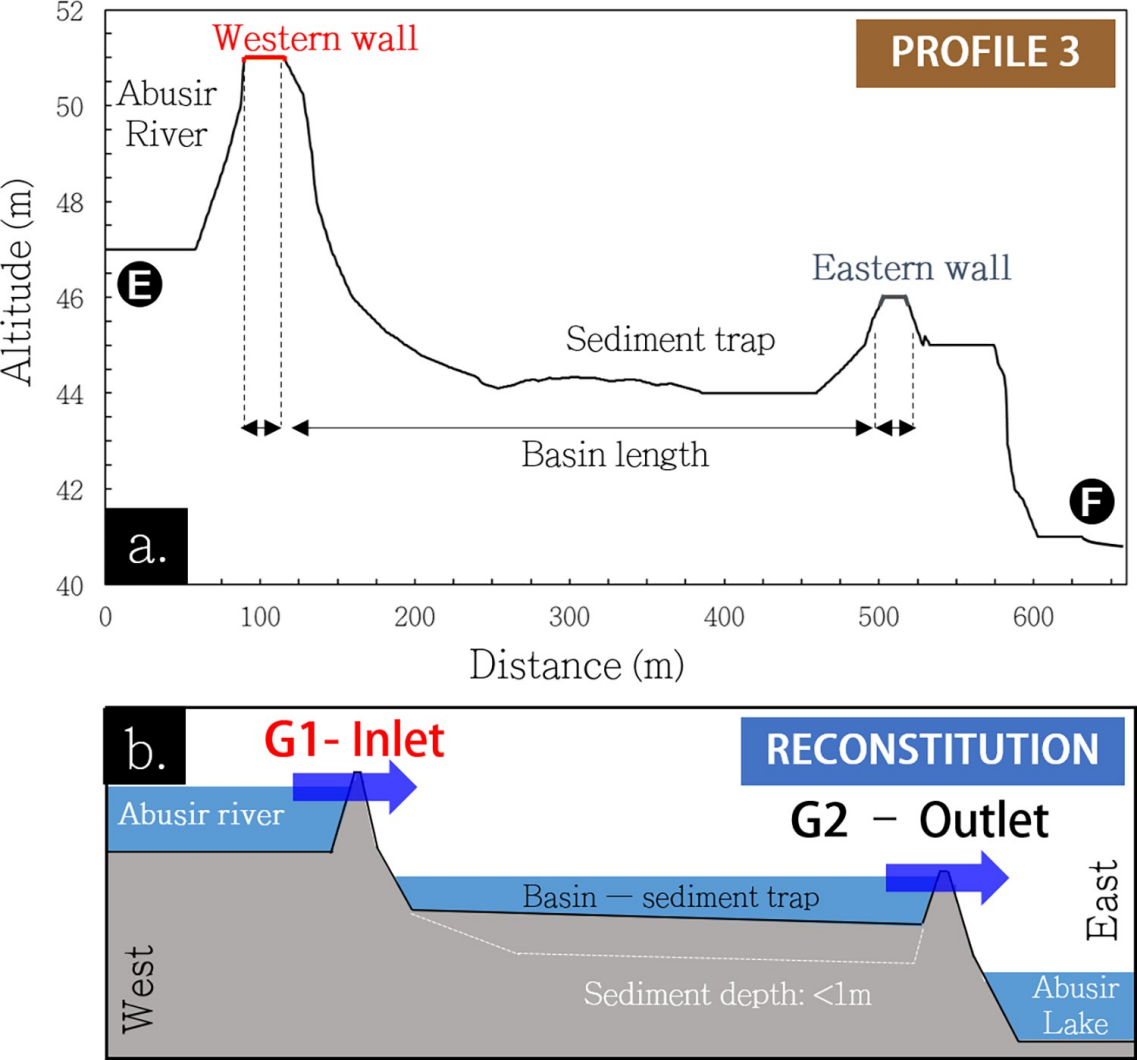
a. West-east elevation profile of the Gisr el-Mudir structure. b: Schematic reconstitution of the profile with water flow.

The excavations performed on the eastern wall of the Gisr el-Mudir highlighted a lower structural quality [45]. Its shape is similar to that of the western wall, with a distinctive parabolic profile (**Fig 6**, line **C-D**). Furthermore, it discloses two topographical singularities: first, its overall altitude is a few meters lower than the western wall (**Fig 7A**). Then, in the southern part of the eastern wall, a geophysics anomaly (**G2** in **Figs 4B** and **Fig 6**) was found to be a series of massive, roughly cut, ‘L’-shaped megaliths [45, 66]. Before our study, these megaliths were thought to possibly be the remains of a monumental gateway–due to their similarities with the Djoser’s complex enclosure’s entrance–but their purpose was not specified [66]. According to our analysis, these megaliths could be the side elements of the water outlets, possibly slit openings [76] that were likely closed off by wood beams but could be opened to drain the basin. They are consistently found near a trench that is 2.2 m deep [45], which we believe is possibly the canal that guided outflowing water. In a nutshell, the eastern wall likely acted as a second check dam to the Abusir flows.

In addition to the two dams formed by the western and eastern walls, the Gisr el-Mudir enclosure forms a basin (**Fig 4**). It is closed to the north by another wall made of limestone blocks, though not very tall (likely <2m) because it is built on a natural ridge [45]. The basin’s southern boundary is also mostly made of a natural ridge. The possible absence of a masonry wall on certain portions on this side was unexplained by previous analyses [45]. However, it makes perfect sense when considering a reservoir function. Anchoring dams against side slopes is indeed the standard approach to guide flows and prevent outflanking [68].

In essence, the Gisr el-Mudir enclosure exhibits the defining features of a check dam (**Fig 7B**). The catchment it intercepts is large enough (15 km^2^), plus eventual water derivation from the Wadi Taflah to produce flash floods transporting significant amounts of gravel, sand, mud, and debris due to its slope during intense rainfalls. The valley upstream of the western wall likely served as a first reservoir where the coarsest gravels tended to deposit. The overflowing water then filled the inner basin of the Gisr el-Mudir, where coarse sand would again deposit. Assuming a storage depth between 1 and 2 meters, the retention capacity of the basin would be approximately 220,000–440,000 m^3^. This volume is in line with the overall water volume of a flash flood that could be produced by the Abusir wadi, which is estimated to be about 75,000–225,000 m^3^, assuming 50 mm of rainfall and a 0.30 runoff coefficient. This key, first structure of the Saqqara hydraulic system would have then delivered clear water downstream in normal time, as well as muddy water with an eventually suspended load of fine sand and clay during rainfall events.

### 3.2 The deep Trench’s water treatment system

#### 3.2.1 General configuration

The Djoser’s Complex is surrounded by a vast excavation area, commonly referred to as the ’Dry Moat’ since Swelim spotted its outlines [77, 78] (**Fig 3**, blue strip). The Dry Moat is alleged to be a continuous trench cut in the bedrock, up to 50 m wide and ≈3 km long, enclosing an area of ≈600 m by ≈750 m around the Djoser complex [77, 79, 80]. When considering an average depth of 20 m for the four sides of the trench [61], the total excavated volume is estimated at ≈3.5 Mm^3^, approximately ten times the Step Pyramid‘s volume. Due to the thick cover of sand and debris [61] accumulated over the past millennia, its precise geometry is incompletely characterized. The moat’s east and south channels are particularly debated [61].

**Fig 8:**
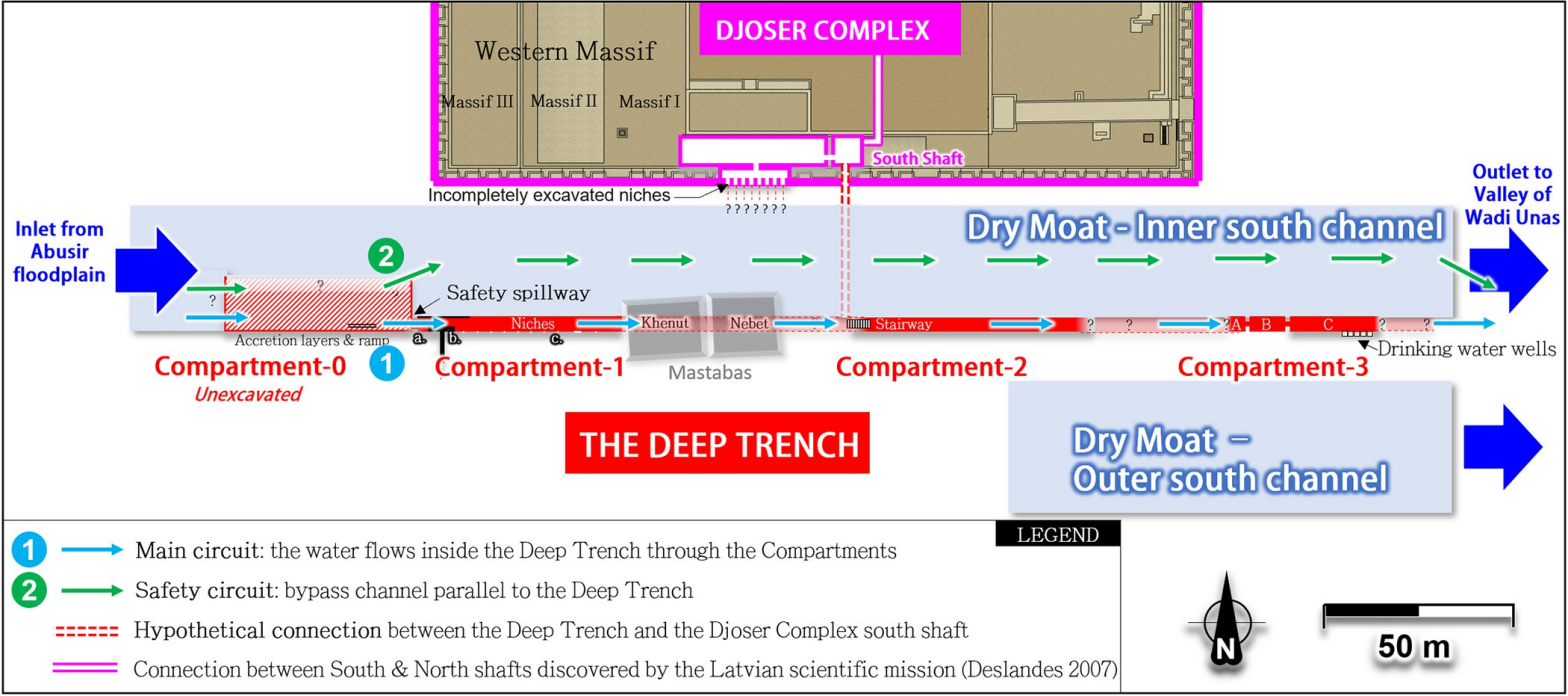
View of the Deep Trench area (red parts) south of Djoser’s complex. Water from the Abusir Lake can follow two parallel circuits.

According to Swelim, the moat’s south channel probably split into two parts, known as the *Inner* and *Outer south channels [78]* (**Fig 8**, blue strips). The Inner south channel is relatively shallow (5–7 m deep), 25–30 m wide, and spans approximately 350 m parallel to the southern wall of Djoser’s complex.

The ‘Deep Trench’ [81] (**Fig 8,** red rectangles and dotted lines) is built inside the Inner south channel, along its south wall. It is a ≈27 m-deep, 3 m-wide, and hundreds of meter-long rock-cut channel with several ‘compartments’. So far, only about 240 m [78] of its probable 410 m length have been subject to archaeological excavations in 1937–1938 [78], 1937–1945 [81], and 1975 [82]. Consequently, approximately 170 m remains unexplored, mainly due to the presence of the later Old Kingdom two groups of mastabas built above the trench and at risk of collapse if submitted to underground excavation (transparent grey parts in **Fig 8**).

Generally, two leading theories are highlighted in the literature to explain the purpose of the trench: (i) a quarry for the Djoser’s complex [47, 83], or (ii) a spiritual function [78, 84, 85]. However, over recent years, authors have pointed out several specificities in the trench’s architectural layout, which seem irrelevant in a religious or mining context [1, 86, 87]. In particular, on the mining aspect, several authors estimate [45, 86] that the form of the track suggests that the extraction of stones was not its sole or even primary function, as it does not match with the ancient Egyptian quarrying methods. Reader also considers that some parts of the trench which are ~27 m deep and covered with a rocky ceiling, are wholly unrealistic for quarrying operations and unlikely to have required the paving found near the trench’s bottom [45]. This point is further emphasized by the narrow width of the excavated Deep Trench (3m), which is impractical in a mining scenario.

On the spiritual aspect, Kuraszkiewicz suggests that the trench may have developed a ritual significance as a gathering place for the souls of the nobles to serve the dead King [86]. Monnier [1] considers that the discovery of several niches in the channel does not fully demonstrate the moat’s religious purpose and considers it secondary. The trench’s ritual significance is also regarded as secondary by Reader [45], who suggests the ritual aspects developed only after the complex’s construction and do not reflect the original function of the structure.

In 2020, based on the archaeological, geological, and climatic evidence, Wong was the first to introduce the idea that the trench may have had a completely different function, being filled with runoff water following downpours [37]. If so, this would explain why it was not until the reigns of Unas and Userkaf (V^th^ Dynasty) that new graves occupied the moat. The onset of drier climatic conditions [31, 88] around the end of the IV^th^ Dynasty would have created more favorable conditions for new constructions inside the moat. Despite the potential impact of Wong’s assumption, it did not receive much attention in the literature. Nonetheless, the current authors believe that Wong’s conclusions make sense when considering Saqqara’s downstream localization of a watershed.

#### 3.2.2 The deep Trench: A series of rock-cut compartments built in a hydrological corridor

The Inner south channel and the Deep Trench are built inside the Unas Valley, a hydrological corridor connecting the Abusir wadi plain to the Nile floodplain (**Fig 3**). Both were thus possibly submitted to (un)controlled flooding *[34, 61]* from the Abusir wadi plain.

**Fig 9:**
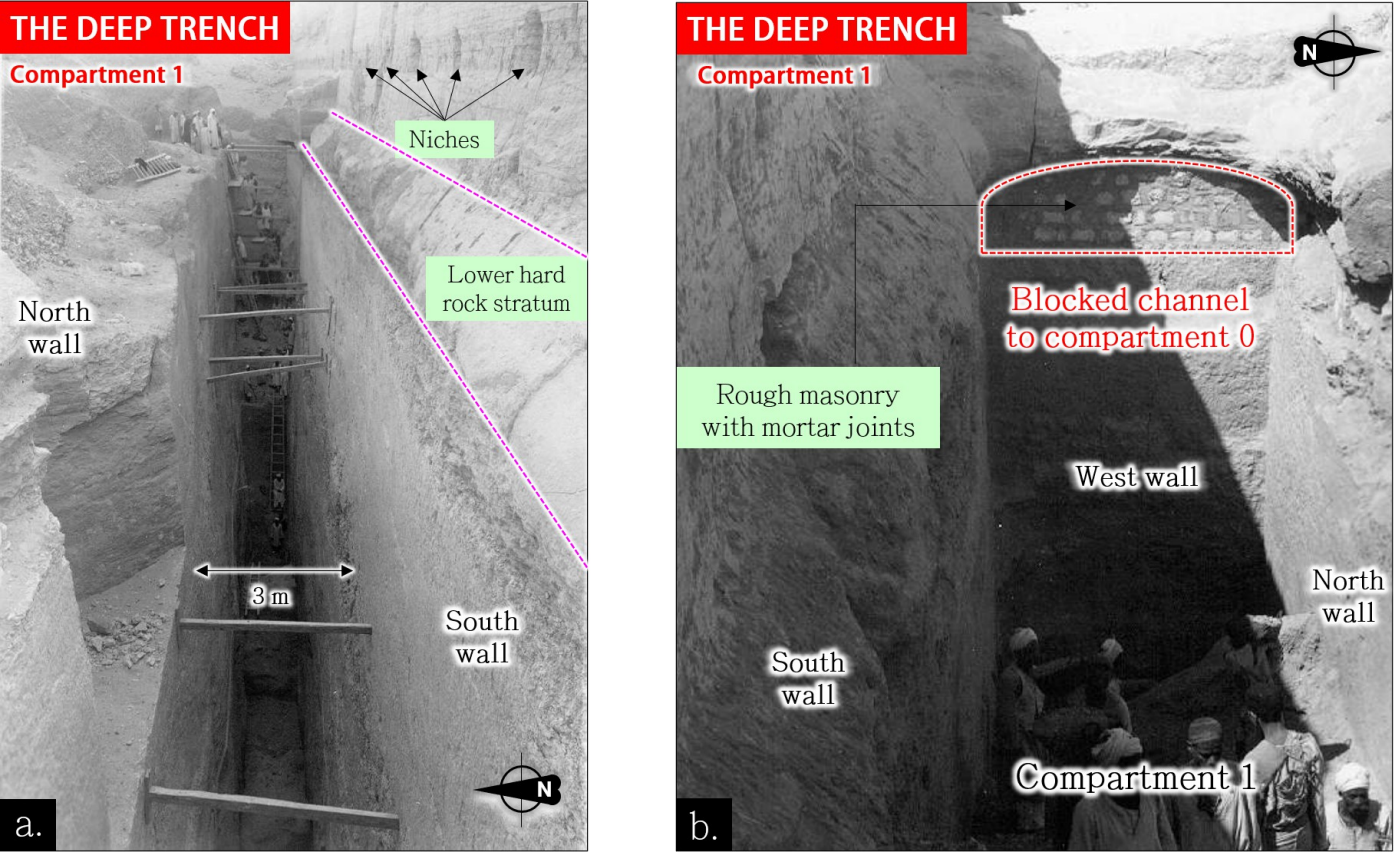
View of compartment-1 of the rock-cut deep trench [78] (1943), 27m deep, 3m wide. a: View from the west; b: View from the east. The workers in the background provide a sense of the structure’s immense scale and technicity.

**Fig 10:**
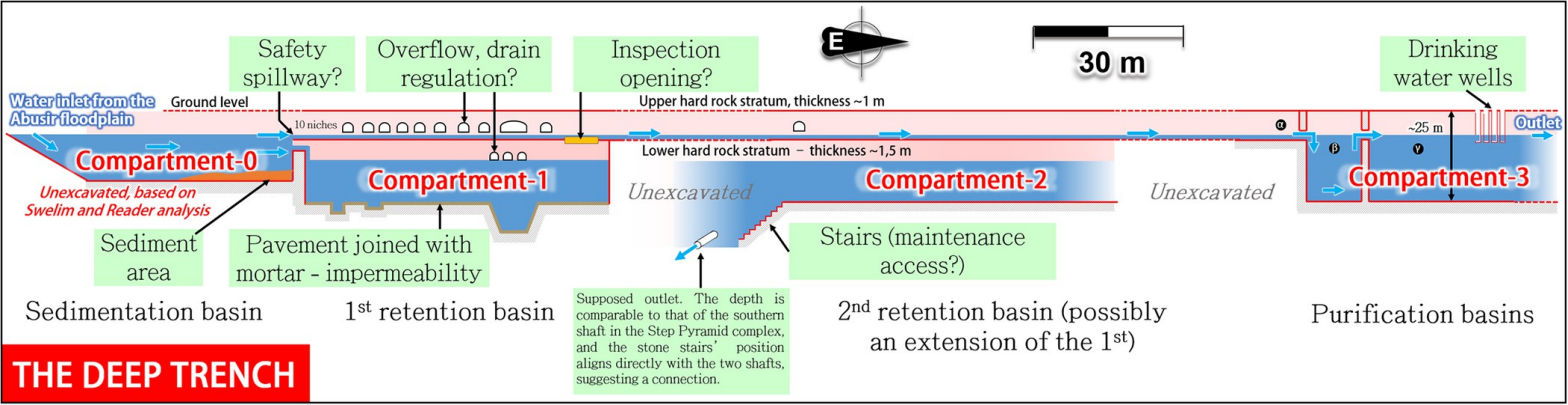
View of the operating structure of the deep trench, found to be a water treatment system. View of the south face.

The Deep Trench connects at least three massive subterranean compartments *[45, 47]* (**Fig 8**, red parts) meticulously carved out with precisely cut surfaces *[78]* (**Fig 9**) and joined by a tunnel *[77]*. A fourth compartment, retroactively named *compartment-0* (**Fig 10**), likely exists *[45, 78].* On a large scale, the perfect geometric alignment of these compartments is remarkable, as well as their parallelism with the Djoser’s complex and their bottom level similar to those of the southern and northern shafts (≈27 m ASL). These spatial relationships have led some authors to consider that the trench was created as a part of Djoser’s Complex *[86, 89, 90].* This assumption has been reinforced by Deslandes’ discoveries of at least two east-west pipes, about 80 m long, connecting the Djoser’s Complex’s subterranean layouts to the Dry Moat’s eastern side *[91].*

Taken together, the Deep Trench architecture highlights technical proficiency and suggests that the ancient Egyptians intended a technical function rather than a spiritual one. Surprisingly, despite the available clues, the Deep Trench has never undergone detailed engineering studies to analyze its features and identify its purpose. The following sections suggest a hydraulic rationale behind the trench’s internal layout (more details in the **Supplement**).

#### 3.2.3 Consistency of the Deep Trench architecture with a water treatment system

Being largely described in the literature *[77, 86, 92]*, the compartments’ layouts are presented in detail in the **Supplement**. Considering its architecture and geographical location, the Dry Moat’s southern section combines the technical requirements of a water treatment system, including sedimentation, retention, and purification. **Fig 10** illustrates a comprehensive outline of the installation’s functioning process. Similarly to the Gisr el-Mudir, we found that the Deep Trench compartments likely served to transfer water with low suspended sediment concentration to the downstream compartments by overflowing. The process of using a series of connected tanks to filter water and remove sediment is an ancient technique that has been extensively documented in archaeological and scientific literature [93–96]. This method has been employed for centuries to clean water and has played a significant role in the development of water treatment practices.

*Compartment-0* presents the minimum requirements of a settling basin (considerable length and width, low entry slope, position at the entry of Unas hydrological corridor) whose purpose is to facilitate the coarse particles’ settling that would overflow from Gisr el-Mudir during heavy rainfalls. The descending ramp along the south wall identified by Swelim *[97]* may have permitted workers to dredge the basin and remove the accumulated sediments along the east wall (**Fig 10)**. The very probable connection *[45, 97]* between compartment-0 and compartment-1, blocked with rough masonry (**Fig 9B** and **S3 Fig** in S2 File), is consistent with an outlet overflowing structure. Additionally, when the flow rate in compartment-0 was too high, the tunnel or even the northern portion of the trench may have been used as a spillway bypass to evacuate excess water toward the eastern portion of the Unas wadi valley (**Fig 8,** safety circuit).

*Compartment-1* is then consistent with a retention basin with > 3000 m^3^ capacity (**Fig 10**, left part). The bottom stone paving with mortar joints probably limited water seepage through the bedrock. Its eastern end could go until the compartment-2 *[45]* to form a single compartment, but this point remains debated *[78, 97]*.

**Fig 11:**
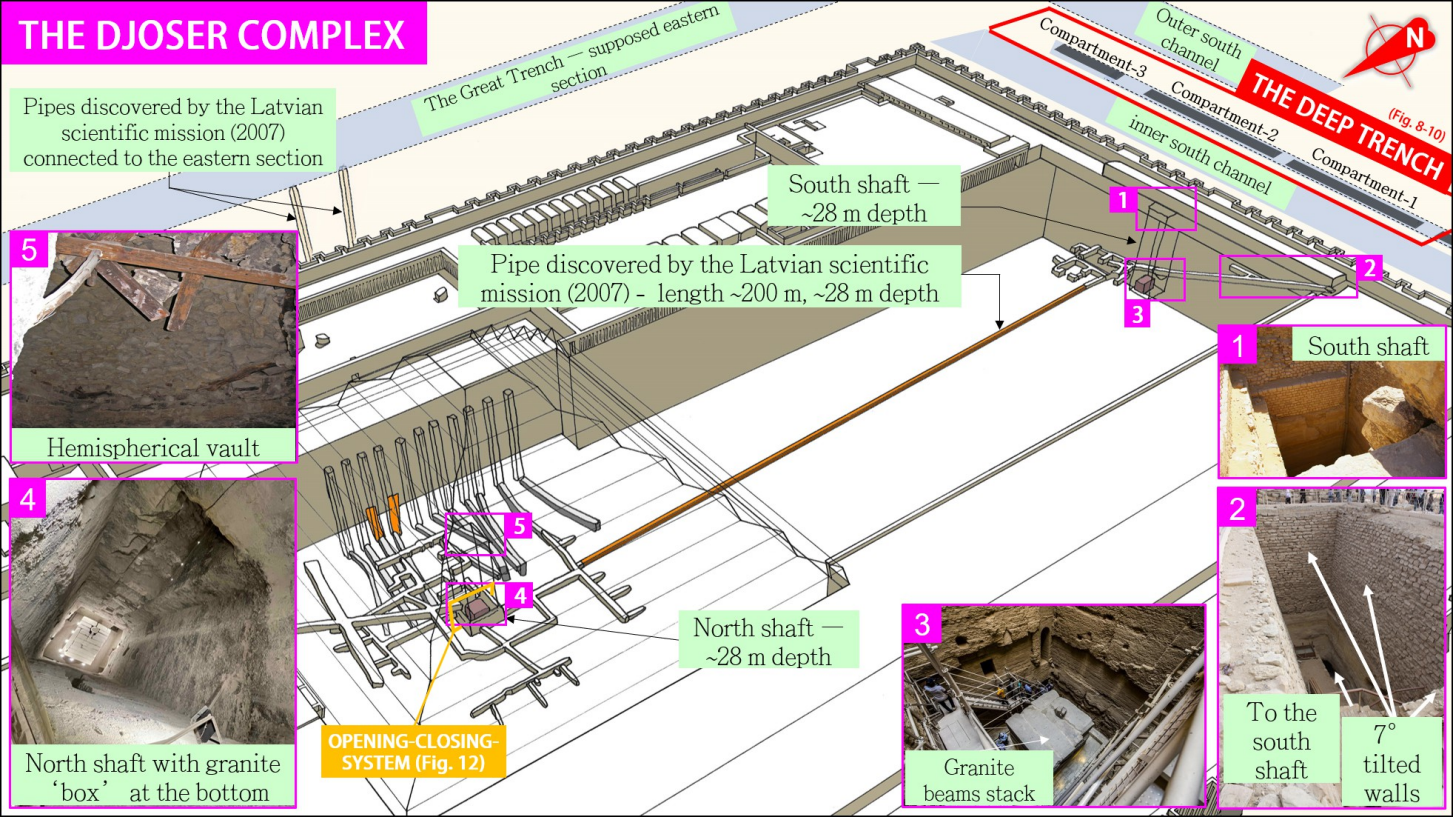
Overview of Djoser’s complex subterranean layout, focusing on the Latvian mission’s discoveries (orange parts)–courtesy of Monnier [1].

*Compartment-2’s* is, unfortunately, largely unexplored (**Fig 10**). Its downstream position might indicate a second retention basin or possibly an extension *[45]* of the first one. The western part of this compartment (stairs area) perfectly aligns with the base levels of the Djoser’s complex south and north shafts, which points towards a connection between the three *[86]*. If so, it would be aligned with the recently discovered pipe of a 200 m-long tunnel linking the bottom of Djoser’s Complex’s southern and northern shafts *[91]* (see next section, **Fig 11**). Compartment-2 would then be another, or an extended, retention basin equipped with a water outlet toward the north.

*Compartment-3* (**Fig 10**, right part) is likely a side purification basin for drinking water. Its position as an appendix of the primary water circuit connecting Gisr el-Mudir to Djoser’s complex seems optimal to minimize water circulation and maximize water-settling time, thus increasing its purification. The second and third sections likely allowed further settling of particles and would have served as reservoirs during dry periods. The relatively smooth walls of the whole structure would have hindered the growth of microbes, plants, and other contaminants, thereby helping maintain the water’s cleanliness *[98]*. Four surface wells allowed access to the end of the last compartment where the water, kept clear and fresh in the shadow of this subterranean monumental cistern, could be used by the building site workforce *[99]*.

The excavated volume of the Deep Trench is greater than 14,000 m^3^ *[77, 86, 92].* If we assume that most of the water available in the Wadi Taflah was diverted toward Saqqara, this volume could be filled about a dozen to more than one hundred times per year on average. We hypothesize a typical filling level of 45 m ASL in the Deep Trench, but an accurate topographical survey is lacking, and the maximum water level could vary between 40–52 m ASL, according to the surrounding terrain elevation.

In essence, we discovered and highlighted for the first time that the Deep Trench’s position and design are consistent with possible use as a water treatment and storage system capable of cleaning and storing thousands of cubic meters of water.

## 4. The central hydraulic lift system

### 4.1 Overview of the Djoser’s complex’ substructure

The internal and external architecture of the Djoser’s Complex is thoroughly documented [1, 3, 100, 101]. The **Supplement** provides an overview of this structure. Basically, the six-step Step Pyramid itself stands slightly off-center in a rectangular enclosure toward the south and reaches a height of approximately 60 m (**Fig 11**). The pyramid consists of more than 2.3 million limestone blocks, each weighing, on average [2], 300 kg, resulting in a total estimated weight of 0.69 million tons and a volume of ≈330,400 m^3^.

The substructure features at least 13 shafts, including two significantly sizeable twin shafts located at the north and south of the complex (**Fig 11**, insets 3&4), and an extensive and well-organized network of galleries descending up to 45 m below ground level [102]. The north shaft is surrounded by four comb-shaped structures distributed on each side and angled 90° apart. Ground Penetrating Radar (GPR) revealed that the twin shaft layouts are connected *[91, 102]* by a 200 m-long tunnel. Moreover, at least two of the twelve shafts on the pyramid’s east side are connected to the supposed eastern section of the Dry Moat by two 80 m long pipes (**Fig 11** and **Supplement**).

From our 3D models, we estimate that ancient architects extracted more than 30,000 tons of limestone from the bedrock to dig the whole underground structure. The total length of the tunnels and subterranean rooms combined is ~6.8 km. However, its layout and purpose remain primarily poorly known and debated [6].

### 4.2 The connected twin shafts

**Fig 12:**
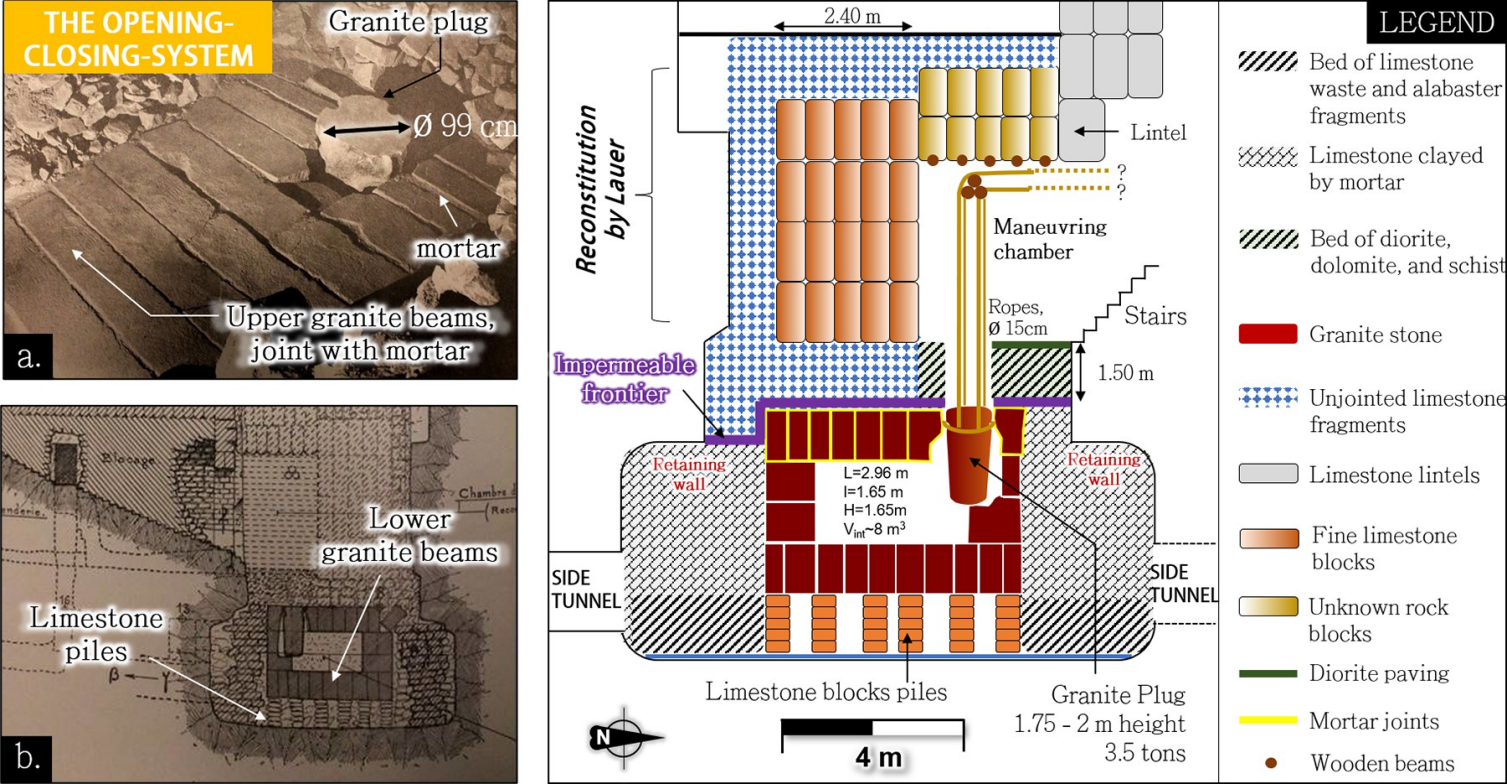
a.: Granite box of Djoser’s complex north shaft serving as an opening-closing system for the water flow coming from side tunnels -source: [113]. b.: Limestone piles supporting the box - source: [3]. c: Diagram of the North Shaft plug system. Redrawn from Lauer sketches [108].

The ‘north shaft’ is located under the pyramid of Djoser and is almost aligned with its summit. This shaft is ≈28 m deep and has a square shape with 7 m sides. Its bottom part widens to ≈10 m on the last, deepest 6 m, forming a chamber (**Fig 12** and **S6 Fig** in S2 File). On its upper part, the shaft extends above the ground level by at least four meters inside the Step Pyramid in the shape of a hemispherical vault that was recently reinforced (**Fig 11**, inset **5**). This upper part inside the pyramid body remains unexplored. However, as noticed by Lauer, the shaft sides above ground level display comparable masonry to that of the southern shaft, indicating a possible upward extension [3]. On the pyramid’s north side, a steep trench with stairs provides access to the shaft.

The ‘south shaft’ is located ~200 m south of the north shaft, close to the Deep Trench (**Fig 11**, inset **1**). Its dimensions and internal layout are broadly similar to the north shaft’s. The substructure of the south shaft is entered through a west-facing tunnel-like corridor with a staircase that descends about 30 m before opening up inside the shaft. The staircase then continues east and leads to a network of galleries whose layout imitates the blue chambers below the Step Pyramid. As mentioned earlier, a 200 m-long tunnel connects the lower part of the north and south shafts (**Fig 11**, orange pipe). A series of deep niches located on the south face of the south shaft [97], the shape of which resembles that of the Deep Trench’s compartments 1 and 2, might indicate a former connection between both. This point remains to be confirmed by additional investigation.

The south shaft is connected to a rectangular shaft to the west via a tunnel-like corridor with a staircase that descends approximately 30 meters before opening up into the south shaft (**Fig 11**, inset **2**). At the corridor level, a chamber has been cut into the bedrock parallel to the descending passage [3], towards the south. This chamber features several incompletely excavated niches on its south wall, which could extend under the south wall of the Djoser complex (**Fig 8**). Pending further excavations, they might indicate a connection with the Deep Trench.

### 4.3 The twin shafts’ internal layout: two plug-systems topped with maneuvering chambers

The initial purpose of the twin shafts’ granite boxes has been largely debated [15, 100]. The presence of two shafts with two similar granite boxes and almost identical substructures was previously explained as a separation of the body and spirit of Djoser [100]. However, the Pharaoh’s body is actually missing and was not found during modern excavations. Several authors and explorers excluded the possibility of King Djoser’s burial in the north shaft [15, 103]. Vyse claimed [15] that the box’s internal volume was too narrow for moving a coffin without breaking the body. Firth and Quibell considered [103] the fragments found by Gunn and Lauer [104] to be of mummies of ‘late date’, possibly belonging to the Middle or New Kingdom. Finally, a thorough radiocarbon dating [105] on almost all retrieved remains [104, 106, 107] located near the granite box excluded the possibility that ‘*even a single one of them*’[105] could have belonged to King Djoser. Therefore, although the northern shaft had clear funerary significance much later, its original purpose during the time of Djoser may have been different.

Unfortunately, the main part of the materials that filled the twin shafts was removed during past archaeological excavations, mainly in the 1930s [108], leaving only the two granite boxes at their bottom (**Fig 11**, insets **3** and **4**). Therefore, the shafts’ internal layout description is mainly based on the explorers’ archaeological reports and testimonies[109–111].

The two granite boxes are broadly similar in shape and dimensions. Both are made of four layers of granite blocks and present top orifices closed by plugs that weigh several tons (**Fig 12A**). The southern box is slightly smaller, with a plug made of several pieces, making it less versatile. The north box does not lay directly on the underlying bedrock but is perched on several piles of limestone blocks supporting the lower granite beams (**Fig 12B**), tentatively attributed to robbers by Lauer [3]. The space around the box is connected with four tunnels arranged perpendicularly on each side of the shaft (see **Supplement**). This space was filled with several successive layers [108] (**Fig 12C**, grey parts). The lowermost layer consisted of coarse fragments of limestone waste and alabaster, making it permeable. Meanwhile, the upper layer, going up to the box ceiling’s level, was made of limestone jointed with clay mortar [108], *i*.*e*., less permeable [112]. This ceiling was itself covered by a 1.50 m thick layer of alabaster and limestone fragments plus overlying filling (**Fig 12C**, blue part), except around the plug hole, which was encircled by a diorite lining, a particularly solid rock (**Fig 12C**, green part).

Directly above the granite boxes were ‘maneuvering chambers [108]’ that enabled the plug to be lifted. The plug closing the north shaft’s box has four vertical side grooves, 15 cm in diameter, intended for lifting ropes (**Fig 12C**) and a horizontal one, possibly for sealing. Below the chamber ceiling and just above the orifice, an unsheathed wooden beam was anchored in the east and west walls (**Fig 12C**). This beam likely supported ropes to lift the plug, similar to those found in the south shaft with friction traces [108].

Interestingly, the granite stones forming the granite box ceiling were bounded by mortar (**Fig 12A**), creating an impermeable barrier with the shaft’s lower part and leaving the plug’s hole as the only possible connection between the shaft and the inside of the box. Conversely, most joints between the box’s side and bottom stones, connected with the permeable bottom layer, were free from mortar.

These details, thoroughly documented during Lauer’s excavation *[3, 108]* and visible on pictures (**Fig 12A** and 12B), clearly point to technical rather than symbolic application. Taken together, the granite box’s architecture and its removable plug surrounded by limestone clay-bound blocks present the technical signature of a water outlet mechanism.

When opened, such a plug system would have allowed the north shaft to be filled with water from the Deep Trench or, in another scenario, from the Dry Moat’s eastern section. The permeable surrounding filling would have permitted water discharge control from the four side tunnels. Then, the water could only seep through the granite box’s lower joints. This design would have prevented water from rushing through the system at high speed and with pressure shocks.

Considering water coming from the Deep Trench (elevation delta: 10–20 m), the retaining walls and the many layers’ cumulated weight stacked over the granite box acted as a lateral blockage and would have prevented the box ceiling from being lifted due to the underlying water pressure.

### 4.4 Consistency of the internal architecture of the Djoser’s complex with a hydraulic lift mechanism

**Fig 13:**
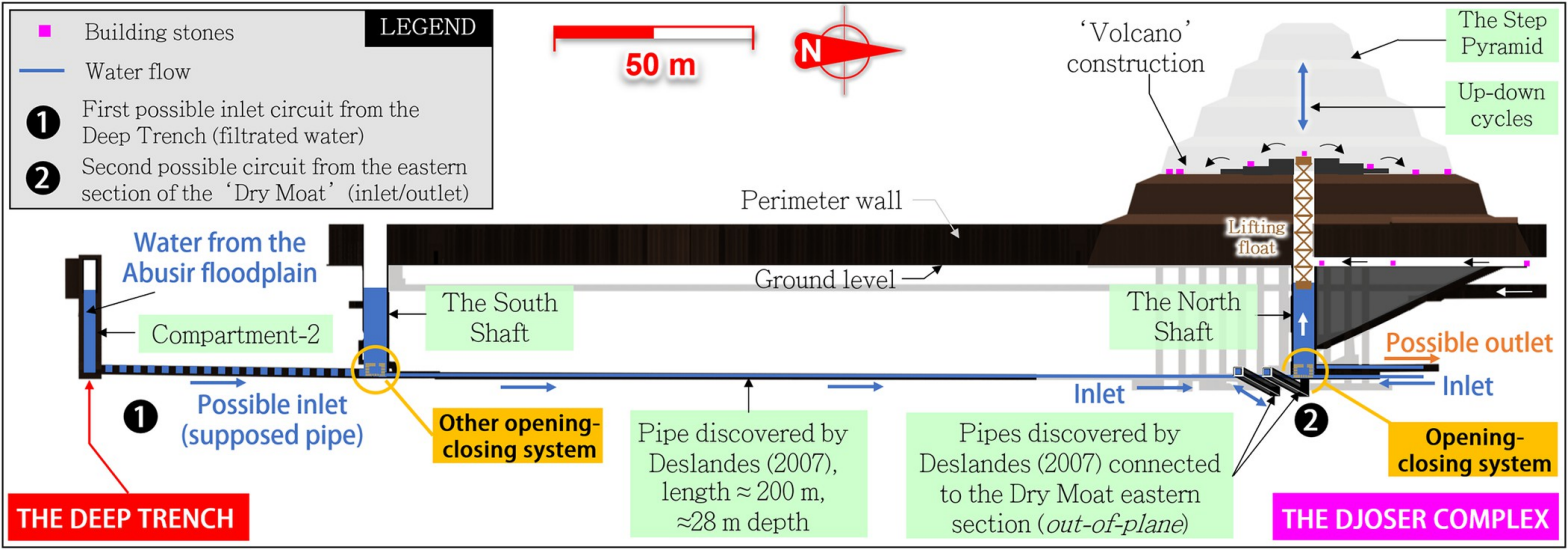
The identified building process of the step pyramid: A hydraulic lift mechanism.

After gathering all the elements of this study, we deduce that the northern shaft’s layout is consistent with a hydraulic lift mechanism to transport materials and build the pyramid. Elements at our disposal indicate that the south and north shafts could be filled with water from the Dry Moat. A massive float inside the north shaft could then raise stones, allowing the pyramid’s construction from its center in a ‘volcano’ fashion (**Fig 13**).

Although a connection between the *Compartment*-2 and the Djoser shafts has yet to be identified, it is highly probable that sediment-free water from the Deep Trench was used in this system (**Fig 13,** disk ‘**1**’). This water quality would have reduced the risk of fouling and malfunction because it minimizes the presence of sand and clay that feed into the north shaft. This would prevent the deposition and progressive filling in the tunnels and connections, as well as the clogging of the joints between the bottom and side granite blocks of the box. The 200 m-long underground pipe [91] that connects the north and south shafts is then consistent with the transfer of water from the Deep Trench’s water treatment system to the north shaft, possibly via the south shaft.

Furthermore, there is a proven connection between the tunnels surrounding the north shaft and the Dry Moat through the Deslandes’ pipes [91] on the eastern side of the complex (**Figs 11** and **13**). Pending further investigation, we hypothesize that the water inlet was located to the south (**Fig 13,** disk ‘**1**’), with the outlet(s) sending water toward the east through two juxtaposed pipes (disk ‘**2**’). Several horizontal galleries connected to these two pipes were acacia-cased [3], a technique commonly used to safeguard the walls in hydraulic works in ancient Egypt. A large stone portcullis [108] found in one of these galleries may have served as a versatile gate closed during the water filling of the north shaft.

In another scenario, the Deslandes’ juxtaposed pipes (**Fig 13,** disk ‘**2**’) could be considered as a water inlet for unfiltered water.

Finally, we hypothesize that a hydraulic lift, a massive float that was possibly made of wood and weighed several tons (see **Supplement**), should run slowly inside the shaft to prevent instabilities and friction with the sides. The stones could have been elevated by filling and emptying cycles, allowing the lift to move up and down with stones (**Fig 13**). These stones could have passed along the northern entrance until the central shaft. Recent discoveries have shown that this gallery was kept open until the very end of the pyramid’s construction, after which it was closed [1, 91]. In our scenario, the stones could have been transported directly at ground level, corresponding to the pyramid’s first course, or slightly higher through a ramp penetrating in a (currently sealed) corridor some meters above the ground level. This configuration would have had the particular advantage of minimizing the elevation gain for which the hydraulic lift would be required. The stones could have been transported via the so-called ‘Saite gallery [114]’ in a final scenario. Although Firth [114] considers this gallery to postdate the III^rd^ Dynasty, it remains possible that it was recut on the basis of an earlier gallery.

### 4.5 Modelling the hydraulic lift mechanism

We developed a simple numerical model of the hydraulic lift to study its water consumption and loading capacity (see **Supplement**). The model was kept as simple as possible to be easily checked and only intended to give relevant orders of magnitudes.

**Fig 14:**
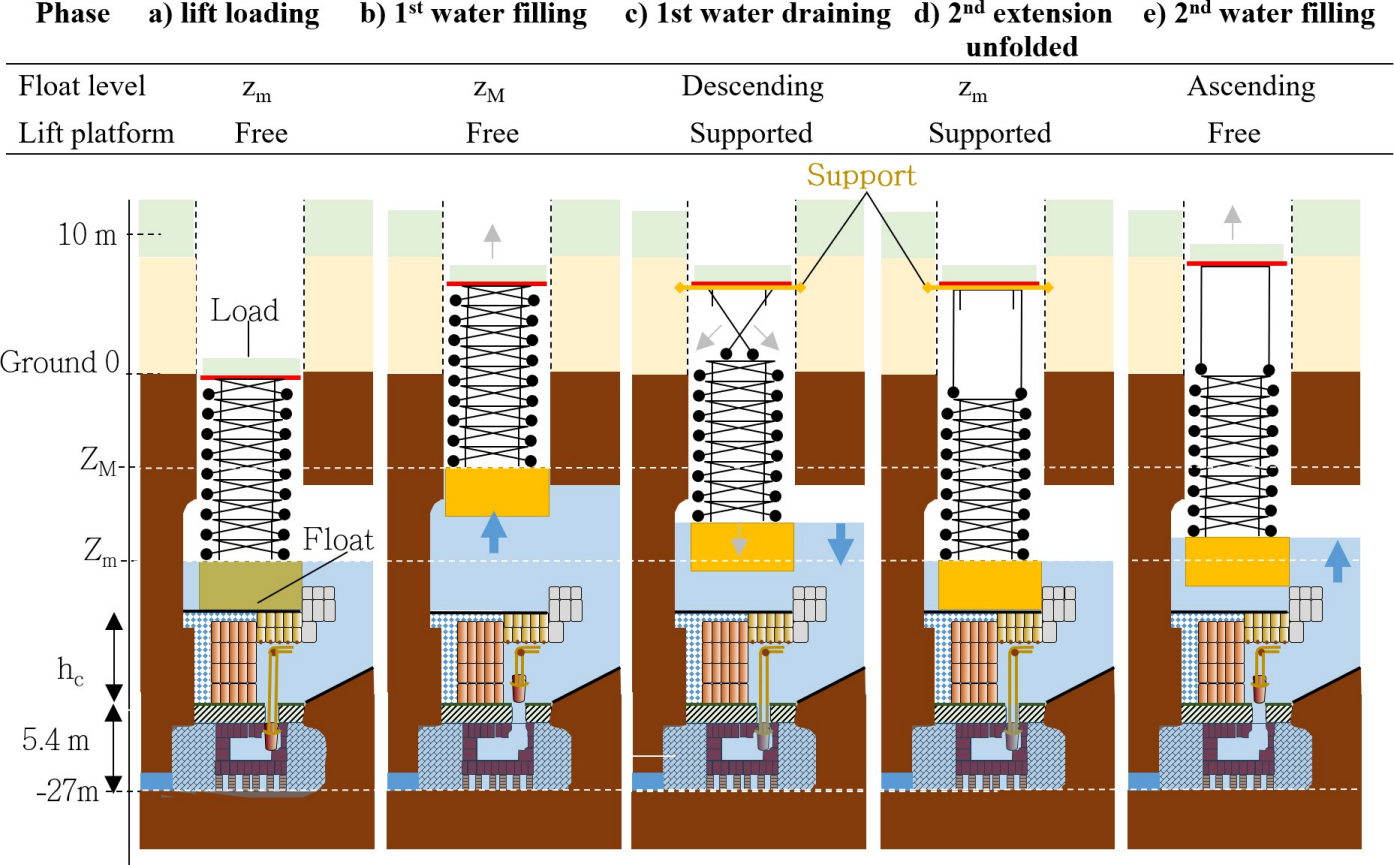
Sketch of the hydraulic lift principle. The lift platform (red line), and extension support (orange line) during the unfolding of the lower element are represented. The associated holes are to be localized in further excavation of the upper part of the shaft.

The hydraulic lift is modelled as a float loaded with stones to build the pyramid and with a vertical extension to raise this material at the necessary level. Based on the initial altitude of the lift, Z_m_, which cannot be below 17m from ground level (the bottom of the shaft was filled with the box and overlying rocks, see **Fig 12C**), and assuming a loading of the material on the lift at the ground level, the maximum height that can be reached in one cycle is <17m. To achieve greater heights, we hypothesize that the lift platform was blocked during the float descent, *e*.*g*., using beams (see **Fig 14**). This modification would have allowed the platform to reach higher altitudes by adding or unfolding an extension. For the top of the pyramid, the float could be conversely used as a counterweight when descending, pulling on ropes that would haul the platform after passing over pulleys above the shaft head. A dual-use method involving hauling during shaft draining and elevating during water filling would have been the optimal management approach.

The beginning of the pyramid building was most probably performed using ramps prolonging the path from the local quarry, possibly the Dry Moat [44]. To provide an upper bound of water consumption, we modelled the pyramid building using the hydraulic lift from the first layer at ground level. Our model suggests that this upper bound value is 18 Mm^3^ of water required to build the whole pyramid using the float to lift stones only when the shaft is filled (see **Supplement**). A few million were required to build the first 20 m and could be saved if ramps were used instead. The total amount of water needed would have been reduced by about one-third if the float had been used as a counterweight, pulling on ropes to haul stones on a platform suspended in the top part of the shaft rather than being located on a wooden frame extension attached to the float. Finally, if both lifting (when filling the shaft) and hauling (when draining the shaft) were used, the water consumption would decrease by two-thirds. If the loading was not performed at ground level but rather through a ramp and gallery above ground level, about one-quarter of the water would be saved if, for instance, using a 5 m-high ramp and 43% for a 10 m-high ramp. Further investigation above the vault and on the pyramid sides could help to identify such an eventual gallery. If, conversely, the loading was performed about 13 m below ground level in the top part of the northern gallery (**S6 Fig** in S2 File), the water consumption would typically increase by two-thirds.

On the other hand, through our research and calculations, we have determined that the Wadi Taflah catchment had the capacity to supply 4–54 Mm^3^ over 20–30 years of construction, therefore not enough when assuming only pessimistic values (lower bound for rainfall and runoff coefficient, fast construction and sub-optimal use of the lift just using it when water rose), but sufficient when assuming intermediate values, and eight-times enough water to meet this demand when assuming optimistic values (upper bounds of parameters and dual lifting-hauling functioning). If further research demonstrates that the higher clay and silt content possibly present at that time shortly after the Green Sahara period probably led to increased runoff coefficients by a factor of 2–3 or even more, the resource would be increased by the same factor.

The climatological conditions on the Saqqara plateau during the III^rd^ Dynasty are still not well understood [37]. As a first assumption, we estimate that the water supply may have been continuous even without an upper Abusir lake’s permanent existence, thanks to the flow from the wadi Abusir and, more significantly, through a probable derivation system from the nearby Wadi Taflah, assuming this large catchment had a more perennial runoff regime. Pedological investigations would be worthwhile in the plateau area and in the talweg of both wadis to look for evidence of more frequent water flow.

As a result, the hydraulic mechanism may have only been usable when sufficient water supply was available, so it may have only been used periodically. Other techniques, such as ramps and levees, were likely used to bring the stones from the quarries and adjust their positions around the lifting mechanism or when it was not in operation.

## 5. Discussion

### A unified hydraulic system

Based on a transdisciplinary analysis, this study provides for the first time an explanation of the function and building process of several colossal structures found at the Saqqara site. It is unique in that it aligns with the research results previously published in the scientific literature in several research areas: hydrology, geology, geotechnics, geophysics, and archaeology. In summary, the results show that the Gisr el-Mudir enclosure has the feature of a check dam intended to trap sediment and water, while the Deep Trench combines the technical requirements of a water treatment facility to remove sediments and turbidity. Together, these two structures form a unified hydraulic system that enhances water purity and regulates flow for practical uses and vital needs. Among the possible uses, our analysis shows that this sediment-free water could be used to build the pyramid by a hydraulic elevator system.

By its scale and level of engineering, this work is so significant that it seems beyond just building the Step Pyramid. The architects’ geographical choices reflect their foresight in meeting various civil needs, making the Saqqara site suitable for settling down and engaging in sedentary activities, such as agriculture, with access to water resources and shelter from extreme weather conditions. This included ensuring adequate water quality and quantity for both consumption and irrigation purposes and for transportation, navigation, or construction. Additionally, after its construction, the moat may have represented a major defensive asset, particularly if filled with water, ensuring the security of the Saqqara complex [115].

The hydraulic lift mechanism seems to be revolutionary for building stone structures and finds no parallel in our civilization. This technology showcases excellent energy management and efficient logistics, which may have provided significant construction opportunities while reducing the need for human labor. Furthermore, it raises the question of whether the other Old Kingdom pyramids, besides the Step Pyramid, were constructed using similar, potentially upgraded processes, a point deserving further investigation.

Overall, the hydraulic lift could have been a complementary construction technique to those in the literature for the Old Kingdom [8, 10]. Indeed, it is unlikely that a single, exclusive building technique was used by the ancient architects but that a variety of methods were employed in order to adapt to the various constraints or unforeseen circumstances of a civil engineering site, such as a dry spell. Therefore, the beginning of the pyramid building was most probably performed using ramps prolonging the path from the local quarry. According to petrographic studies [47], the main limestone quarry of the Saqqara site could correspond to the Dry Moat that encircles the Djoser Complex, providing access on the four sides of the pyramid for the extracted blocks and reducing the average length of the ramps.

### An advanced technical and technological level

By their technical level and sheer scale, the Saqqara engineering projects are truly impressive. When considering the technical implications of constructing a dam, water treatment facility, and lift, it is clear that such work results from a long-standing technical tradition. Beyond the technical aspects, it reflects modernity through the interactions between various professions and expertise. Even though basic knowledge in the hydraulics field existed during the early Dynastic period, this work seems to exceed the technical accomplishments mentioned in the literature of that time, like the Foggaras or smaller dams. Moreover, the designs of these technologies, such as the Gisr el-Mudir check-dam, indicate that well-considered choices were made in anticipation of their construction. They suggest that the ancient architects had some empirical and theoretical understanding of the phenomena occurring within these structures.

### …questioning the historical line

The level of technological advancement displayed in Saqqara also raises questions about its place in history. *When these structures were built remains the priority question to answer*. Were all the observed technologies developed during the time of Djoser, or were they present even earlier? Without absolute dating of these works, it is essential to approach their attribution and construction period with caution. Because of the significant range of techniques used to build the Gisr el-Mudir, Reader estimates *[70]* that the enclosure may have been a long-term project developed and maintained over several subsequent reigns, a point also supported by the current authors. The water treatment facility follows a similar pattern, with the neatly cut stones being covered and filled with rougher later masonry. Finally, the Djoser Step Pyramid also presents a superposition of perfectly cut stones, sometimes arranged without joints with great precision and covered by other rougher and angular stones [3]. Some of these elements led some authors [6, 100] to claim that Djoser’s pyramid had reused a pre-existing structure.

### Some remaining questions

The Deep Trench was intentionally sealed off at some point in history, as evidenced by the pipe blockage between Compartment-0 and Compartment-1. The reasons are unknown and speculative, ranging from a desire to construct buildings (such as the Khenut, Nebet, or Kairer mastabas) above the trench to a technical malfunction or shutdown due to a water shortage. This sealing might also have been done for other cultural or religious purposes.

The current topography of the land around the Djoser complex, although uncertain given the natural or anthropogenic changes that have occurred over the last five millennia, does not support the existence of a trench to the east side. Therefore, our observations join those of Welc et *al. [61]* and some of the first explorers [63], reasonably attributing only three sections to the Dry Moat.

## 6 Materials and methods

- High-resolution commercial satellite images (Airbus PLEIADES, 50 cm resolution) and digital elevation models (DEM) were computed and analyzed to identify Abusir wadi’s palaeohydrological network impact on Djoser’s construction project. The processing sequence to generate DEM was mainly achieved using the *Micmac* software *[116]* developed by the French National Geographic Institute (IGN) and the open-source cross-platform geographic information system *QGIS 3*.*24*.*3*. *Tisler*.
- Geospatial data analysis was performed using the open-source WebGL-based point cloud renderer Potree 1.8.1 and *QGIS 3*.*24*.*3*. *Tisler*.
- The 2D CAD profiles of the Step Pyramid Complex presented throughout this article were produced using *Solidworks 2020 SP5 (Dassault Systems)*, *Sketchup Pro 2021 (Trimble)*, *Blender (Blender Foundation)*, and *Unreal Engine 5 (Epic Games)*, mainly based on dimensions collected by successive archaeological missions during the last two centuries reported in the literature.
- The Wadi Taflah watershed and the catchment area west of Gisr el-Mudir have been identified and characterized using *QGIS 3*.*24*.*3*. This was done with the help of the *Geomeletitiki Basin Analysis Toolbox* plugin, developed by *Lymperis Efstathios for Geomeletitiki Consulting Engineers S*.*A*. based in Greece.
- The modeling of the hydraulic lift mechanism was performed using the open-source programming software *RStudio 2022*.*07*.*2*.

## 7. Concluding remarks and perspectives

**Fig 15:**
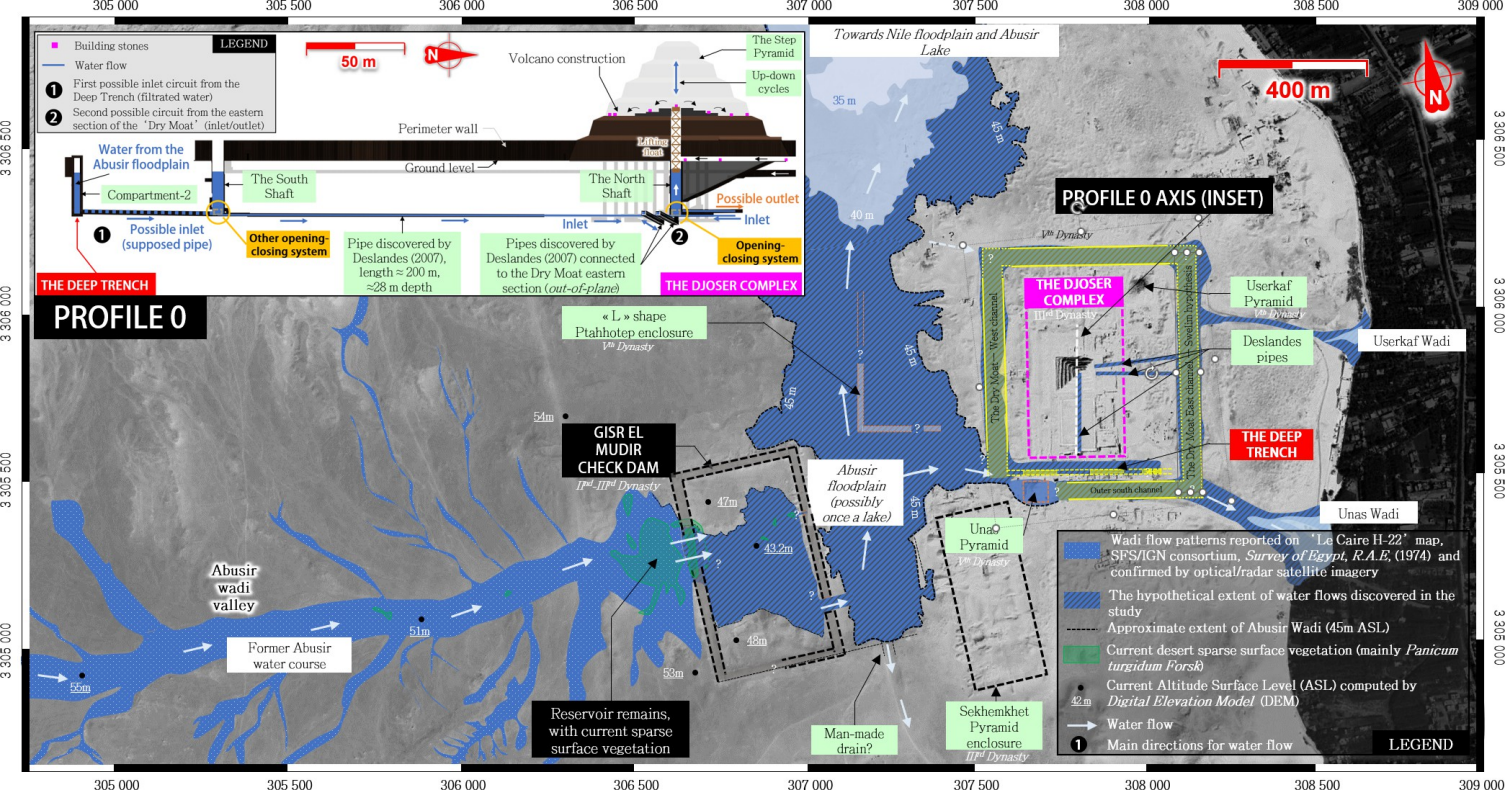
Summary of the study. North Saqqara map showing the relation between the Abusir water course and the Step Pyramid construction process (Inset). The arrows figuring the flow directions are approximate and given for illustrative purposes based on the Franco-Egyptian SFS/IGN survey [52]. Satellite image: Airbus Pléiades, 2021-07-02, reprinted from Airbus D&S SAS library under a CC BY license, with permission from Michael Chemouny, original copyright 2021.

This article discloses several discoveries related to the construction of the Djoser complex, never reported before:

1. The authors presented evidence suggesting that the Saqqara site and the Step Pyramid complex have been built downstream of a watershed. This watershed, located west of the Gisr el-Mudir enclosure, drains a total area of about 15 km^2^. It is probable that this basin was connected to a larger one with an estimated area of approximately 400 km^2^. This larger basin once formed the *Bahr Bela Ma River*, also known as *Wadi Taflah*, a Nile tributary.
2. Thorough technical analysis demonstrates that the Gisr el-Mudir enclosure seems to be a massive sediment trap (360 m x 620 m, with a wall thickness of ~15 m, 2 km long) featuring an open check dam. Given its advanced geotechnical design, we estimate that such work results from a technical tradition that largely predates this dam construction.
  To gain an accurate understanding of the dam’s operating period, the current authors consider it a top priority to conduct geological sampling and analysis both inside and outside the sediment trap. This process would also provide valuable information about the chronological construction sequence of the main structures found on the Saqqara plateau.
3. The hydrological and topographical analysis of the dam’s downstream area reveals the potential presence of a dried-up, likely ephemeral lake, which we call Upper Abusir Lake, located west of the Djoser complex. The findings suggest a possible link between this lake and the Unas hydrological corridor, as well as with the ‘Dry Moat’ surrounding the Djoser complex.
4. The ‘Dry Moat’ surrounding the Djoser complex is likely to have been filled with water from the Upper Abusir Lake, making it suitable for navigation and material transportation. Our first topographical analysis attributes only three sections to this moat (West, North, and South).
5. The Dry Moat’s inner south section is located within the Unas hydrological corridor. The linear rock-cut structure built inside this area, called ‘Deep Trench,’ consisting of successive compartments connected by a rock conduit, combines the technical requirements of a water treatment system: a settling basin, a retention basin, and a purification system.
6. Taken as a whole, the Gisr el-Mudir and the Deep Trench form a unified hydraulic system that enhances water purity and regulates flow for practical uses and vital needs.
7. We have uncovered a possible explanation for how the pyramids were built involving hydraulic force. The internal architecture of the Step Pyramid is consistent with a hydraulic elevation device never reported before. The current authors hypothesize that the ancient architects could have raised the stones from inside the pyramid, in a volcano fashion. The granite stone boxes at the bottom of the north and south shafts above the Step Pyramid, previously considered as two Djoser’s graves, have the technical signature of an inlet/outlet system for water flow (**Fig 15**). A simple modeling of the mechanical system was developed to study its water consumption and loading capacity. Considering the estimated water resources of the Wadi Taflah catchment area during the Old Kingdom, the results indicate orders of magnitude consistent with the construction needs for the Step Pyramid.

## Graphical conclusion

Fig 15.

## Supporting information

S1 Fig (PNG)

S1 File (DOCX)

S2 File (DOCX)
