# Supplementary figures and images for: On the possible use of hydraulic force to assist with building the step pyramid of saqqara
Source: PLoS One. 2024 Aug 5;19(8):e0306690. doi: 10.1371/journal.pone.0306690 (PMC11299825; doi:10.1371/journal.pone.0306690)

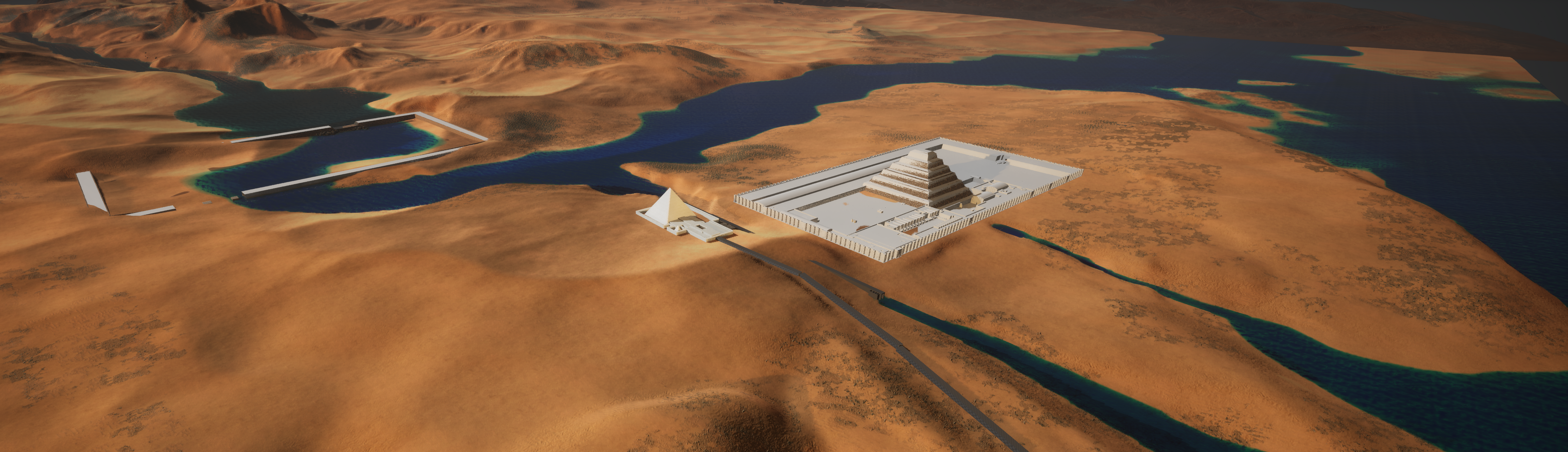

Supplement: S1 Fig — (PNG) [file pone.0306690.s001.png]
