# Supplementary material for: On the possible use of hydraulic force to assist with building the step pyramid of saqqara
Source: PLoS One. 2024 Aug 5;19(8):e0306690. doi: 10.1371/journal.pone.0306690 (PMC11299825; doi:10.1371/journal.pone.0306690)
Supplement: S2 File — (DOCX) [file pone.0306690.s003.docx]

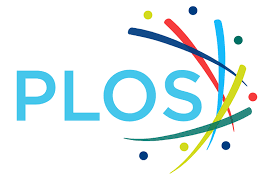


Supporting information for

**On the possible use of hydraulic force to assist with building the Step Pyramid of Saqqara**

Xavier Landreau *et al.*

*Corresponding author. Email: [xavier.landreau@cea.fr](mailto:xavier.landreau@cea.fr) or [paleotechnic@pm.me](mailto:paleotechnic@pm.me)

**This PDF file includes:**

Supplementary Text.

Figs. S1 to S8.

Table S1.

References 1 to 37 refer only to references in the Supporting Information document.

Supplementary Text

1. THE SAQQARA’S HYDROLOGICAL NETWORK
   1. Digital elevation model of the Saqqara area

As discussed in the main paper, we created a *Digital Elevation Model* (DEM - **Fig. S1**) to visualize the topography near Saqqara with greater precision. The processing sequence to generate DEM was mainly achieved using the *Micmac* software *[1]* developed by the French National Geographic Institute (IGN) and the open-source [cross-platform](https://en.wikipedia.org/wiki/Cross-platform) [geographic information system](https://en.wikipedia.org/wiki/Geographic_information_system) *QGIS 3.24.3*. *Tisler.* Using these models, we identified water flows and potential lake formation areas. Delineation and catchment areas’ parameters extracted from this DEM show that the Abusir wadi drainage area is bordered by limestone rock outcrops of an average height of 20-25 m (**Fig. S1**, pink line). In its western part, the catchment area is separated from the Wadi Taflah by less than 1 km. Its current catchment summit is about 110 m ASL, giving the Abusir wadi an average 1% slope until the Gisr el-Mudir (around 45 m ASL).

Downstream of the Gisr el-Mudir, the topographical shape is quite flat and open, it could be considered typical of a zone where an ephemeral lake or wetlands could have existed. Unfortunately, the thick sandy layer covering the area [2] creates topographical uncertainties, and further geological investigations are necessary to draw definitive conclusions.

- 1. Radar confirmation of the existence of the Wadi Taflah watershed

To provide further details on the fluvial activity related to the Wadi Taflah watershed, we used the data from the *Phased Array type L-band Synthetic Aperture Radar* (PALSAR – see **Fig. S2**), which is a remote-sensing instrument on the Japanese *Advanced Land Observing Satellite* (ALOS). PALSAR is an active microwave sensor that operates at L-band frequency, enabling observation of land day and night in any weather conditions. The radar's L-band (1.25 GHz) can penetrate several meters of low electrical loss material, such as aeolian sand, thereby probing the first subsurface geological features in arid regions [3, 4]. Previous studies [5] have shown that L-band orbital SAR could reveal the Sahara's buried and previously unknown paleo-drainage channels.

According to **Fig. S2**, the radar drainage patterns west of Saqqara are similar to the one observed by optical means, with three branches easily recognizable (see orange inset). These patterns confirm the existence of the Wadi Taflah paleohydrological network and its old age.

As mentioned in the main paper, we investigated the possibility of water transfer to the Abusir wadi via an artificial or natural channel connected to the surrounding catchments. However, after analyzing the available radar and optical data, we were unable to detect any connection. Nevertheless, the ALOS radar sensor’s resolution is about 50 m, which limits the detection to major paleoflows. Therefore, a geophysical investigation would be needed to confirm the existence of a derivation channel.

1. THE DRY MOAT
   1. The Deep Trench: summary of past discoveries over the last century

The following paragraphs provide additional information regarding the ‘Deep Trench’ compartments:

- *Compartment-1*: According to the descriptions provided by Saad *[6]*, three aligned niches are hewn on the compartment’s south wall (see **Fig. S3**). These niches are considered ~2 m high, separated by a ~2.5 m interspace. A rocky duct topped the compartment, with a possible opening close to Khenet’s mastaba. Remarkably, the duct’s south wall displays a second row of 10 niches parallel to the first row and about 9 m above. These are all similar (~3 m high and wide) except for the ninth, which is larger (~4.5 m). The niches’ horizontal depth is unknown, and their purpose has yet to be identified. Although religious functions have been suggested [7], we cannot exclude them as pipe entrances or holes for beams.
- *Compartment-2:* this compartment lies in the alignment of the preceding in the easterly direction (see **Fig. S4**). It belongs to Saad’s area excavated in 1939-40 over a distance of ~80 m between Nebet and Kairer mastabas. The compartment’s width is 3 m, and its depth reaches ~25 m from the ground level and ~16 m if we count from the lower hard rock stratum’s level. The compartment’s walls were found to be generally vertical with cutting and surface flatness quality similar to those of compartment-1. At the base of compartment-2’s western end, Zaki Saad found [6] several stone stair-steps that went down to an even deeper level. However, no additional clearing was done since the fill at the western end of Saad's clearance looked to be sustaining the late Fifth Dynasty mastaba of Queen Nebet. At ground level and 8-9 m depth, Saad identified several projecting rocky outcrops corresponding to the lower and upper hard rock strata. In all likelihood, these outcrops are the remains of two destroyed parallel rocky ceilings above compartment-2. Because of the alignment, it is generally admitted that the rock duct above compartment-1 extended over compartment-2 *[8]*. Furthermore, a niche of similar shape and dimensions was identified in the south wall of the duct and is still visible today. Interestingly, this and other niches are broadly similar to those in the Djoser complex’s south shaft’s south wall.
- *Compartment-3:* it comprises three parallelepiped chambers designated as α, β, and γ (see **Fig. S5**). These chambers are interconnected by two orifices located near their top. Each room is separated from its neighbor by approximately 5 m. The chamber α is blocked on its western end by a filling. The excavated chambers β and γ reach a depth of up to 25 m. The geophysical investigation conducted by Herbich [9] revealed that the compartment extends beneath the rubble at the eastern end of chamber γ. On the south wall of this chamber, excavators discovered ten wells cut in the rock, four of which have an opening inside the γ room.
- *Compartement-0:* its existence is supported by Saad's discovery [6] of accretion layers west of compartment-1, below Unas’ Pyramid. These stone layers run ~14 m along the south rock wall and extend into the bedrock, sloping by a few degrees. The masonry of these layers seems similar to that used to block the void, suggesting a connection between compartments 0 and 1 [2]. According to Swelim [8], this masonry closely resembles the accretion layers of the Step Pyramid’s grand stairways and recalls the south shaft’s narrowing corridor or even the Sekhemkhet entrance [10]. Such layers were generally built slanting outwards at open pits or inwards when supporting passages. Unfortunately, we do not have complete information about its geometrical characteristics due to insufficient excavations. However, based on pictures and views of the site taken by Saad [6], we estimate that the compartment had a linear extension beyond Unas' pyramid area, with a length greater than 70 m. From satellite imagery, its section appears generally asymmetrical, and its depth seems to increase as it approaches compartment-1. On the south side, the compartment appears aligned with the other three compartments. On the north side, the compartment-0 seems much wider than the other compartments, possibly connected with the northern part of the inner south channel.

A tunnel likely connected this series of three rocky compartments. This tunnel was dug between two layers of hard rock (the *lower* and *upper hard rock strata*), more than 300 m long. The excavation was made possible by the unique geology of the Saqqara plateau, which features alternating layers of soft and hard rock [11]. It appears as though the Deep Trench was carefully crafted to take advantage of this geological feature *[11]*.

1. THE DJOSER’S PYRAMID SUBSTRUCTURE

The underground structure of the Step’s pyramid has been extensively described and documented in literature [12, 13, 14, 15, 16]. This part aims only to provide visual illustrations of this substructure:

- A north-south cross-section of the Step Pyramid is presented in Fig. S6a. The different colors (rose, blue, green, grey) correspond to the developmental phases that culminated in its step pyramidal form suggested by Lauer [12]. Following Lauer's excavations, the main stages are traditionally labeled Mastaba 1 (M1), Mastaba 2 (M2), Pyramid 1 (P1), and Pyramid 2 (P2).
- An overview of the tunnel network surrounding the Step Pyramid's central shaft is presented in **Fig. S6**b, with the same corresponding colors as mentioned above. The eleven shafts at the pyramid’s east side are ≈33 m deep. According to the Latvian mission’s ground penetrating radar (GPR) analysis [17], two of them are connected to the east side of the Dry Moat (**Fig. S6**b, A & B black disks). These connections are located at the junction between the III &IV vertical shafts and their acacia-cased horizontal galleries. Two other connections might exist (C&D) but are insufficiently excavated. Moreover, a 200 m-long tunnel discovered by GPR analysis [17, 18] connects the north and south shafts’ substructures (E black dot).
- Additionally, we present a 3D reconstitution of the layout of the Step Pyramid's central shaft (**Fig. S7**). The CAD model and textures are based on the information reported by Lauer [12] and realized with *Unreal Engine 5* software (Epic Games).

As a reminder, Mays et *al.* point out that cisterns or drains have historically been utilized for water storage and underground chambers for fugitives, burial sites, and prison cells [19].

1. THE CENTRAL HYDRAULIC LIFT SYSTEM
2. Modelling the hydraulic lift system

A simple mechanical model of the hydraulic lift system was developed to study its water consumption and loading capacity. The model was kept as simple as possible to be easily cross-controlled and only intended to give relevant orders of magnitudes. We consider that the whole system worked on the basis of communicating vessels, *i.e*., interconnected reservoirs that can be drained and re-filled by opening or closing water gates and where water circulates by differences of elevation between the system components (shafts, reservoir, trench). The water from the Gisr-el-Mudir can be transferred to the Deep Trench purification facility before joining the lower part of the north well situated beneath the Djoser pyramid. This water influx could lift the stone-loaded float from the bottom.

The hydraulic lift is modeled in the first scenario as a float holding the material used to build the pyramid and a vertical extension to lift the material to the required height. (see **Figure 14**, main paper**)**. The device operated in Djoser's north shaft, a 7x7m well. The float size is designed almost to fill the shaft laterally to prevent inclination caused by uneven loading during loading or unloading. A float’s square section of side *a =* 6.5 m is used in the following analysis. The float is assumed to be made of wood, such as Lebanese Cedar, a material commonly used in ancient Egypt [20], having a density *ρ_f_* ≈560 kg/m³. The total loading capacity is computed assuming that buoyancy *ρ_•_a²_•_h* with *ρ* = 1000 kg/m³ the water density, and *h* the float height (m), that holds the gravity force applied to the lift:

$\rho\cdot a^{2}\cdot h= \rho_{f}\cdot a^{2}\cdot h+w_{e}+w_{l}$ (1)

With *w_e_* the extension weight (kg) and $w_{l}$the load weight (kg). Note that we consider a pseudo-static condition where acceleration is negligible and that the gravitational acceleration was removed from both sides for conciseness. Rearranging Eq. (1) enables us to deduce the total loading capacity:

$w_{e}+w_{l}=(\rho- \rho_{f})\cdot a^{2}\cdot h$ (2)

This corresponds to 18.6 t/m of float. Therefore, 55 to 90 tons can be elevated by a 3 m-thick or 5 m-thick float, respectively. This weight corresponds to those of the largest blocks found in the pyramids of the Old Kingdom (see **main paper**, introduction). After consulting *Eurocode 5*(29), we determined the feasibility of a wooden extension using four 0.2m x 0.2m beam modules and interconnections. Considering a lift going to the top of the pyramid (62m), the extension weight would reach *w_e_ =*11 tons. A slightly higher float could accommodate a heavier structure if needed.

While acacia and Lebanon cedar wood were not commonly found in ancient Egypt, some quantities could be found in neighbouring countries. When compared to the boats from the Old Kingdom era, the amount required for the Step Pyramid project are reasonable and achievable. To put it into perspective, one of the two Cheops' boats was constructed using Lebanon cedar wood and weighed 45 tons [21] meaning about 90 tons for the two boats.

Instead of using the float and an extension to raise the load when the shaft is filling with water, we can use the float as a counter-weight to pull the load using long ropes and pulleys, or similar systems, at the top of the shaft. With this approach, Eq. (2) is simpler, and the weight that can be hauled is about the same as the weight of the float.:

$w_{l}=\rho\cdot a^{2}\cdot h$ (3)

The bottom of the north shaft is at an altitude of approximately 28 m ASL (*Above Sea Level*), corresponding to 27 m below the ground level (about 55 m ASL). The exact water level of the upstream reservoir used to feed the system and calculate the maximum water level in the shaft, z_M_, is unknown. z_M_ correlates with the water height in the Deep Trench facility, which is linked to the water level and availability downstream of the Gisr el-Mudir.

As described in the main paper, a sophisticated granite box was installed at the bottom of the north shaft. Its upper level is about *h_b =_* 5.4 m above the shaft basement. This box was sealed with a plug and covered with rocky layers, likely to withstand hydrostatic pressure when the shaft was empty. (see **Figure 12**, main paper). The thickness *h_c_* of this cover is not precisely known. Assuming that there is no lateral blockage between the stone cover and the shaft walls – a conservative hypothesis –, it can be written that this cover would be to the point of being lifted by the hydrostatic pressure if it would equal the cover submerged weight. Assuming that hydrostatic pressure was applied to the 1.5 m-thick alabaster bed set above the granite chamber, equalling hydrostatic pressure with the submerged weight of the covers gives:

$\rho\cdot\left( z_{M}-z_{m} \right)=\rho\cdot\left( z_{M}-\left( z_{b}+h_{b} +h_{c} \right) \right)=\left( \rho_{r}-\rho\right)\cdot h_{c}+\rho_{f}\cdot h$ (4)

With *ρ_r_* the covering stones’ density is assumed to be 2600 kg/m³.

$h_{c}=\frac{\rho\cdot\left( z_{M}-\left( z_{b}+5.4 \right) \right)-\rho_{f}\cdot h}{\rho_{r}\cdot}$ (5)

Taking a maximum water level of 45 m ASL and a float height of 3 or 5 m, Eq. (4) leads to a cover height of 3.8 or 3.4 m, respectively, which is consistent with the sketches produced during the excavation of the shaft [13] (see **Figure 12**). The bottom chamber and its covering were thus possibly 5.4 + 3.4 ≈ 8.8 m-high (top-level ≈ 38 m ASL), assuming a 3-m high float. The float lying on this cover would have thus an upper face at 36.8 m ASL below which it was useless to drain the water when operating the lift. The float maximum shift was thus plausibly ≈ 45 – 40.2  ≈ 5 m-high. If the water level in the upstream water feeding systems was higher, up to 52 m ASL, which is lower than the surrounding terrain, the floating course would have been increased by up to 52 – 40.2 = 12 m. This may have motivated the ancient builders to fill and maintain a ponding area between Gisr-el-Mudir and the dry moat with water level as high as possible.

The pyramid construction is modeled by pyramid courses of a given thickness *t =*1 m, with each pyramid course labeled with an index (*i*)*.* For the various pyramid course’s levels *z_i_*, the related surface area A(*z_i_*) is computed knowing the full pyramid shape. The volume of the associated material *V(z_i_)* to be lifted is computed from *V(z_i_) = t • A(z_i_).* The weight *W(z_i_)* is calculated assuming a rock density *ρ_r_ : W(z_i_) = ρ_r_  • V(z_i_)*. The computation of the number of times the lift should be loaded with material *N(z_i_)* is based on the weight of the entire pyramid course and on the lift capacity calculated in Eq. (2) (or in Eq. (3) is rather than assuming hauling, or the sum of both if assuming lifting and hauling):

$N\left( z_{i} \right)=\frac{W\left( z_{i} \right)}{w_{l}}= \frac{\rho_{r}\cdot t \cdot A\left( z_{i} \right)}{\left( \rho- \rho_{f} \right)\cdot a^{2}\cdot h-w_{e}}$ (6)

When lifting material at a height *z* higher than the lift’s entire course, we hypothesize that the load was blocked at its present height by wooden beams sliding in holes on the sides of the pyramid central shaft, or suspended on ropes fixed near the top of shaft. The shaft underground part was drained, making the float go down and leaving room between the load and the float to unfold an articulated extension, most probably made of wood beams, capable of holding a full lift load. To raise the material to the summit of the pyramid, several cycles of shaft filling and draining were necessary to unfold several extension devices that were piled up on the float. As can be seen in **Figure 13**, 3 to 10 m are available between the ground level at which the material was likely loaded on the lift (*z_g_* ≈55 m ASL) and the maximum float level (*z_M_* ≈ 45 – 52 m ASL).

Whatever the shaft’s maximum and minimum water level, *z_M_* and *z_m_*, overall, single load weighting *w_l_* that must be lifted to an elevation *z_i_* from the ground elevation *z_g_* will require the shaft to be filled by water on an equivalent elevation gain *z_i_ – z_g_.* A float course will simply require more cycles of filling and draining.

The cumulated water used to raise an entire pyramid course *i* is thus proportional to the number of loads *N(z_i_),* the elevation gain z_i_ – z_g_, and the volume of water necessary to fill the shaft *V_s_.* This enables us to compute the number of cycles of shaft filling required to lift a pyramid course *N_s_(z_i_)*:

$N_{s}\left( z_{i} \right)=N\left( z_{i} \right)\cdot\frac{\left( z_{i}-z_{g} \right)}{(z_{M}-z_{b_{m}})}$ (7)

The cumulated water used to build the pyramid is finally computed by summing up the water consumption for each pyramid course. Using Eq. (6) in Eq. (7) and summing on each pyramid course gives:

$V_{w}=\sum_{i} N_{s}\left( z_{i} \right)\cdot V_{s}=\sum_{i} \frac{\rho_{r}\cdot t \cdot A\left( z_{i} \right)}{\left( \rho- \rho_{f} \right)\cdot a^{2}\cdot h-w_{e}}\cdot\frac{\left( z_{i}-z_{g} \right)}{(z_{M}-z_{b_{m}})}\cdot V_{s}$ (8)

If one assumes the shaft volume to have a square section of side *c =* 7 m, its volume *V_s_* is simplified by *V_s_ = c² (z_M_ – z_m_)* and Eq. (8) is simplified in:

$V_{w}=\sum_{i} N_{s}\left( z_{i} \right)\cdot V_{s}=\sum_{i} \frac{\rho_{r}\cdot t \cdot A\left( z_{i} \right)}{\left( \rho- \rho_{f} \right)\cdot a^{2}\cdot h-w_{e}}\cdot\left( z_{i}-z_{g} \right)\cdot c^{2}$ (9)

To prepare **Fig. S9,** the model was used with the parameter values provided in **Table S1**.

1. Modelling the pyramid construction

A numerical model was developed to study water consumption, lift cycles, and shaft filling during pyramid construction. The synthetic results are provided in **Fig. S8**. These results show that raising the first pyramid courses (height < 10 m) requires much less water (**Fig. S8**d**)** than the mid-height pyramid courses (20 < height < 40 m) because the elevation gain is limited, although each course is usually > 1 ha against about half less in the mid part. The reduced surface area leads to a significant decrease in required water, while the elevation gain remains substantial.

Finally, building a pyramid to its final height of 62.5 m would require a minimum volume of 18 Mm³, which corresponds to 0.6-0.9 Mm³/yr over a period of 20-30 years. (**Fig. S9**d**)**. These values only provide an initial estimate and do not account for water losses caused by infiltration and other factors.


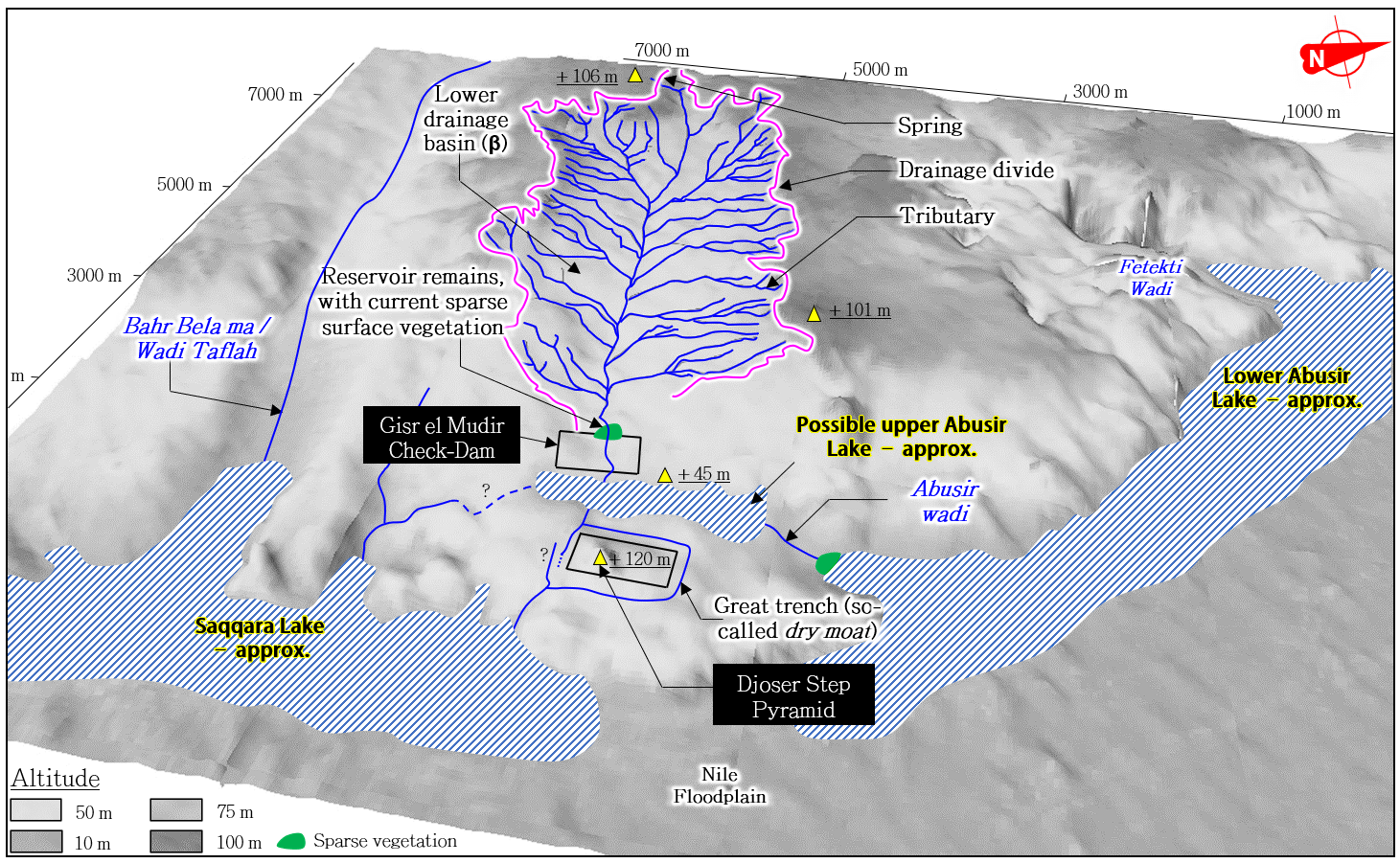


Fig. S1.

ALOS Digital Elevation Model (AW3D30, one arcsec res., 81 km² area) of the Saqqara lower drainage area (β – pink line) and its connections with the Gisr el-Mudir and the Djoser’s.


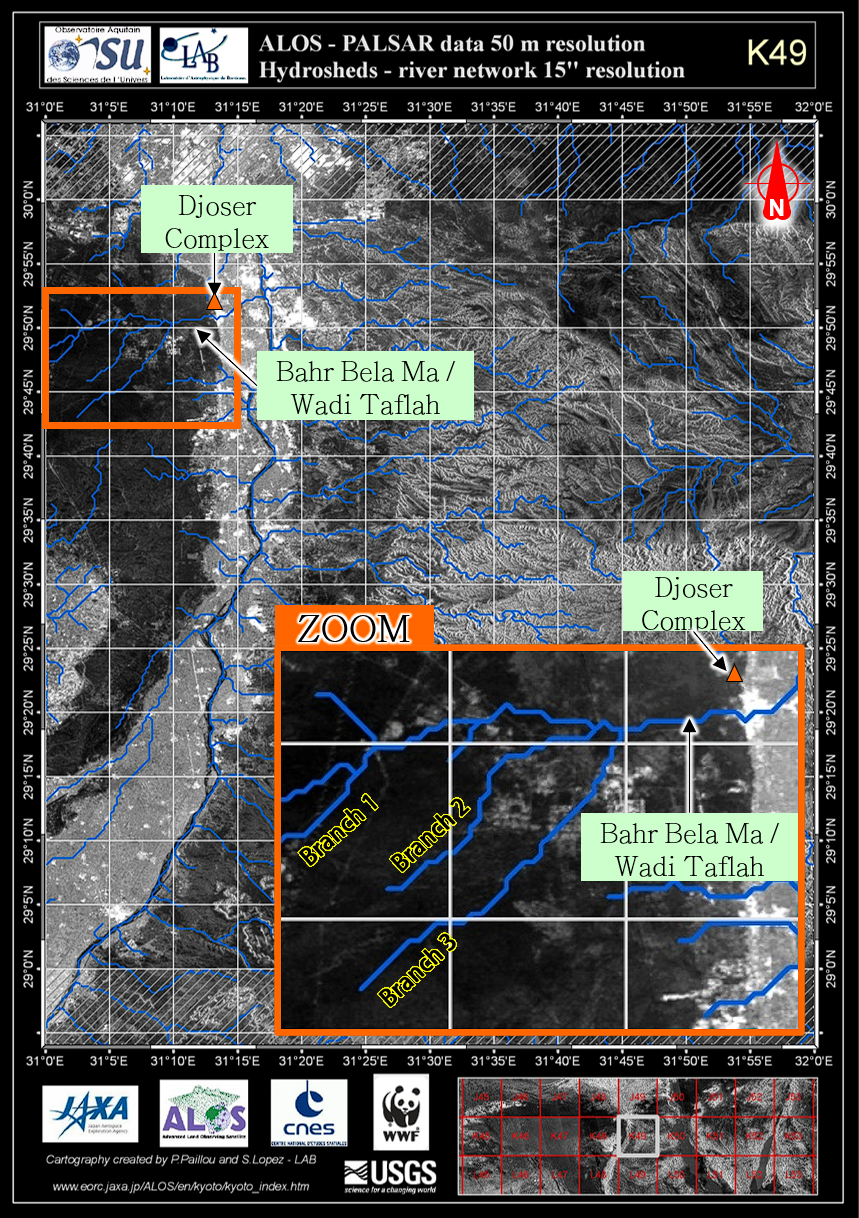


Fig. S2.

L-band (1.25 GHz) ALOS-PALSAR mosaic (50 m resolution) of the discovered paleodrainage system connected to the Bahr Bela ma/wadi Taflah. “Radar paleorivers” appear as bright features due to the accumulation of coarse gravel that produces a higher radar return. The pattern corroborates the surface paleochannels identification on optical imagery.


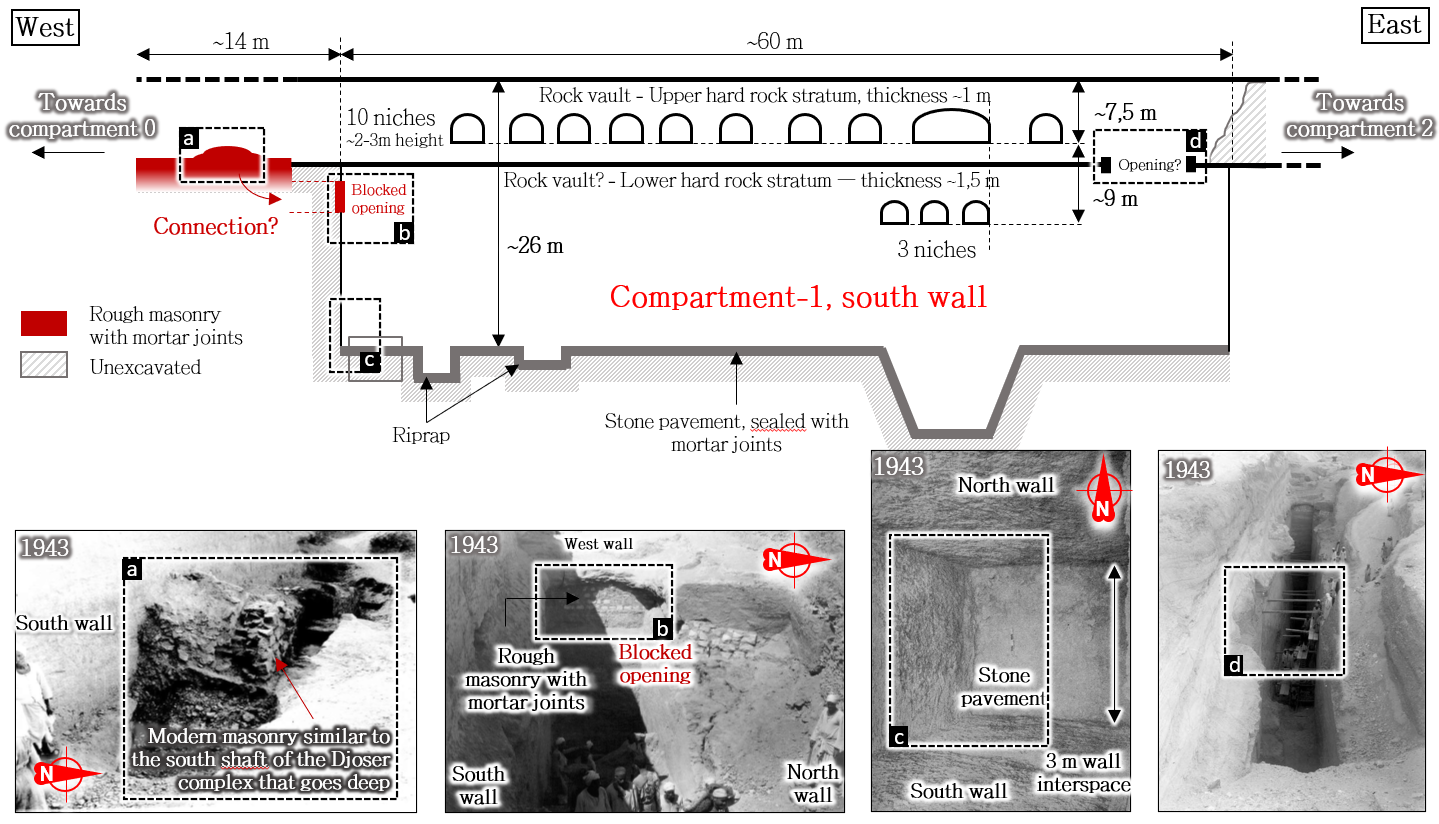


Fig. S3.

South wall diagram of the Deep Trench’s compartment-1.


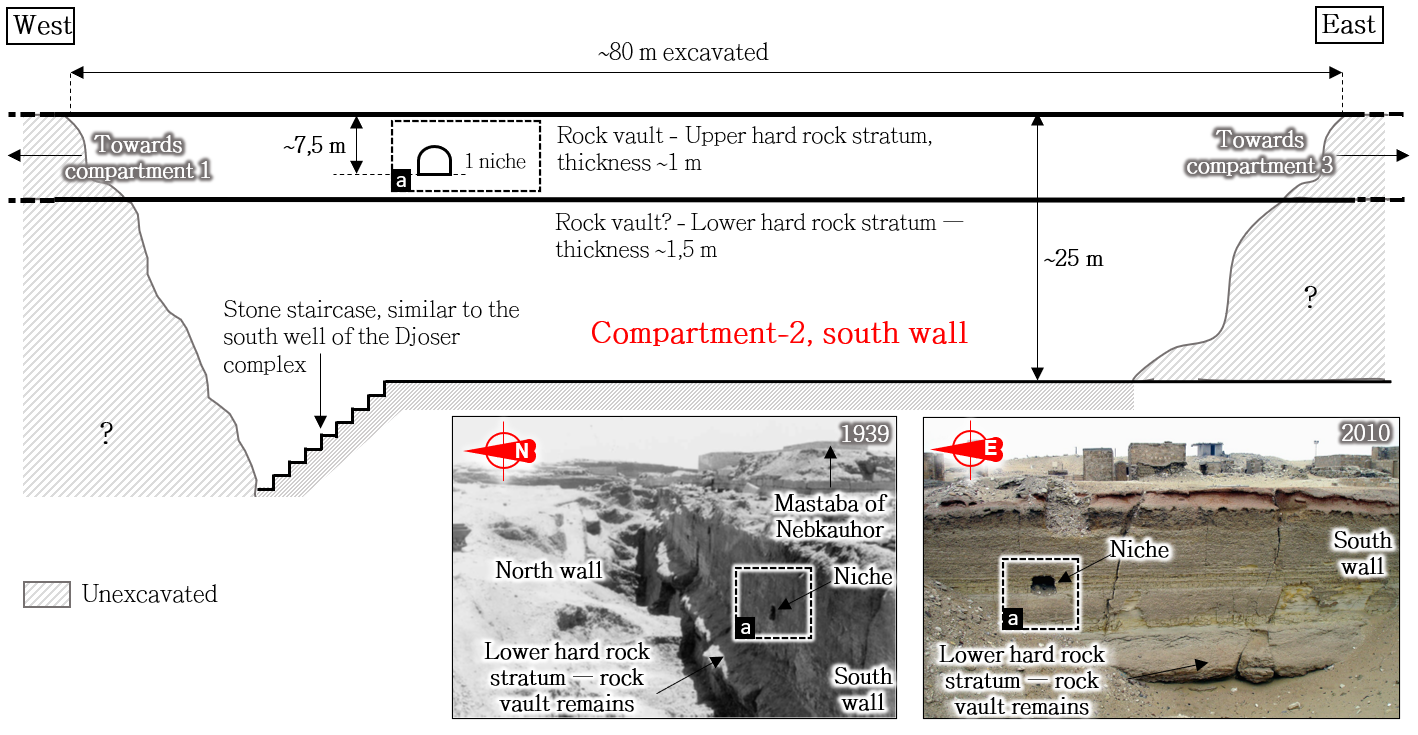


Fig. S4.

South wall diagram of the Deep Trench’s compartment-2.


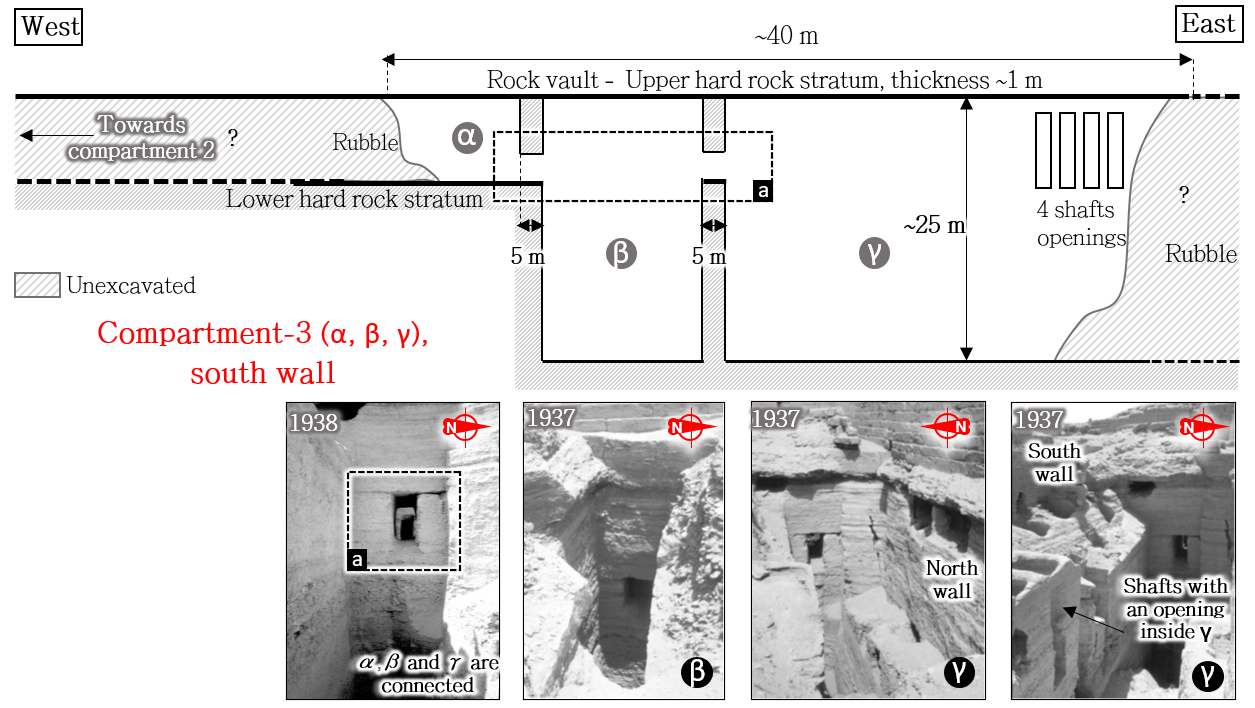


Fig. S5.

South wall diagram of the Deep Trench’s compartment-3.


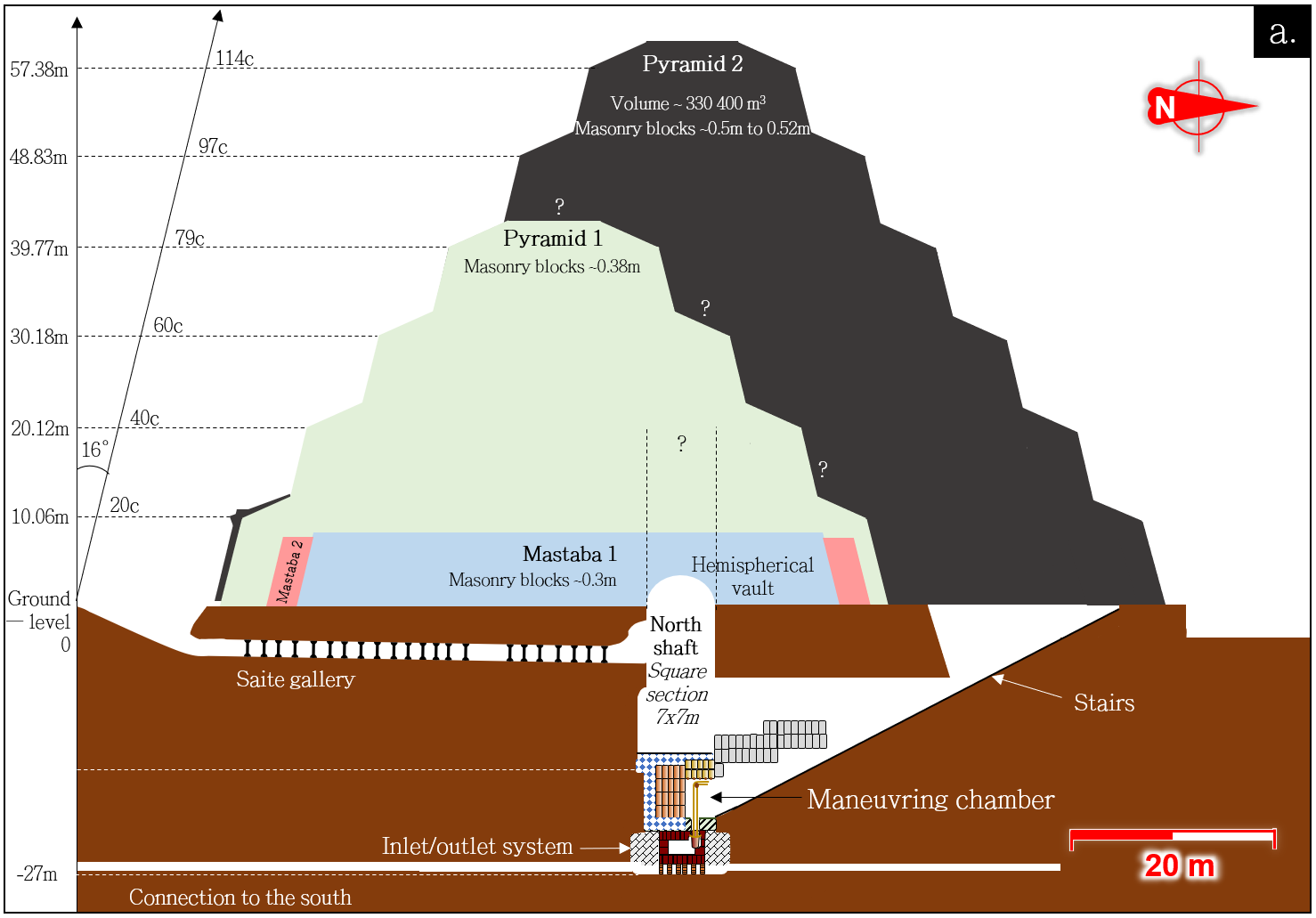

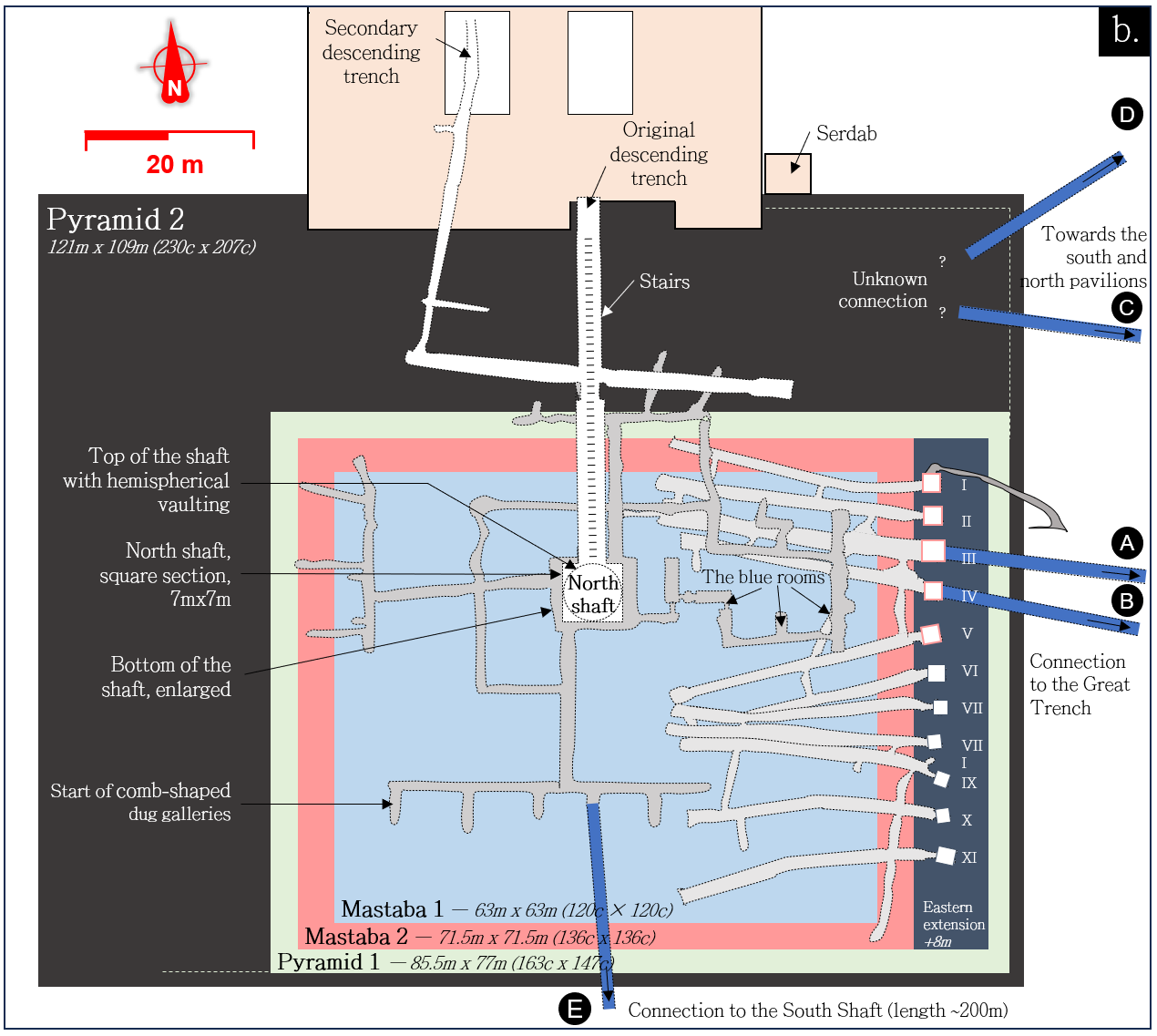


Fig. S6.

a.: North-south cross-section of the Step Pyramid.

b.: Overview of the tunnel network surrounding the Step Pyramid's north shaft. Blue lines display the connections identified by the Latvian mission[17] (2007).


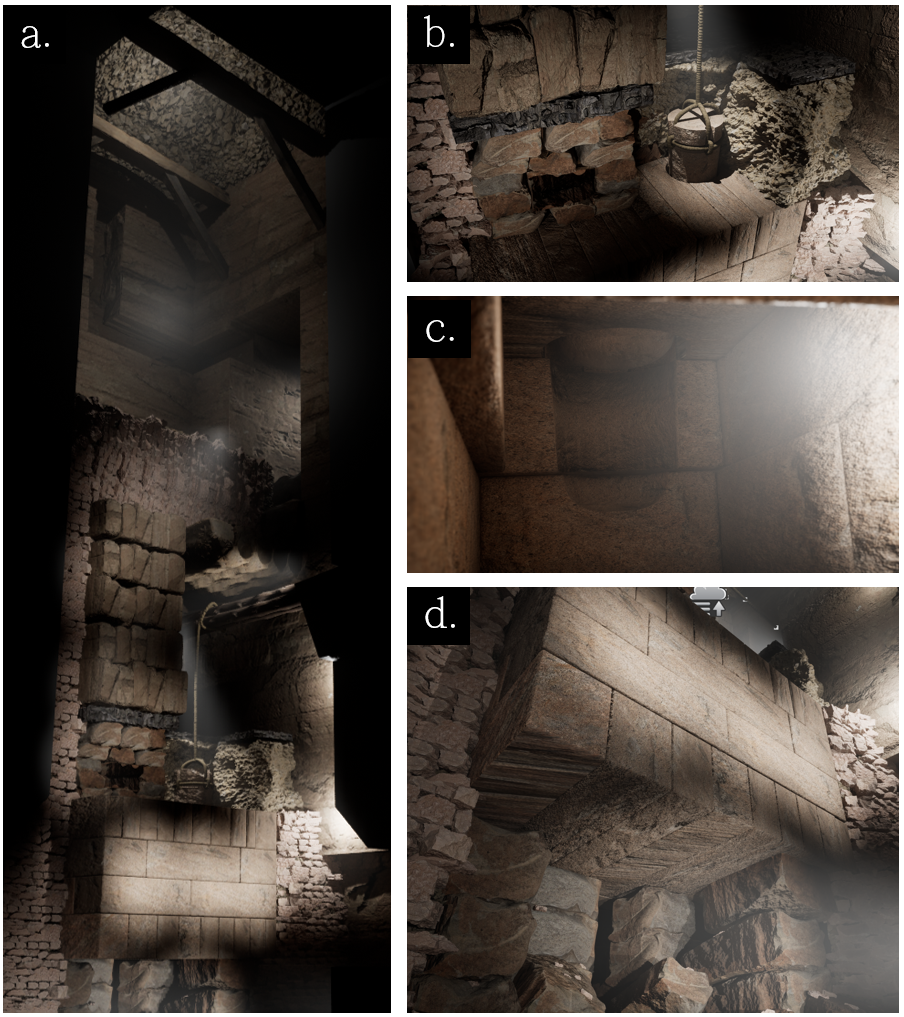


Fig. S7.

*Unreal Engine* 5 (Epic Games) CAD reconstitution of the Djoser’s north shaft layout, according to Lauer [12].

1. Overview of the shaft layout, with the granite box at the bottom, and the maneuvring chamber above.
2. The plug system found to be a water inlet/outlet mechanism.
3. Inside the granite box.
4. View of the stone pills below the granite box.


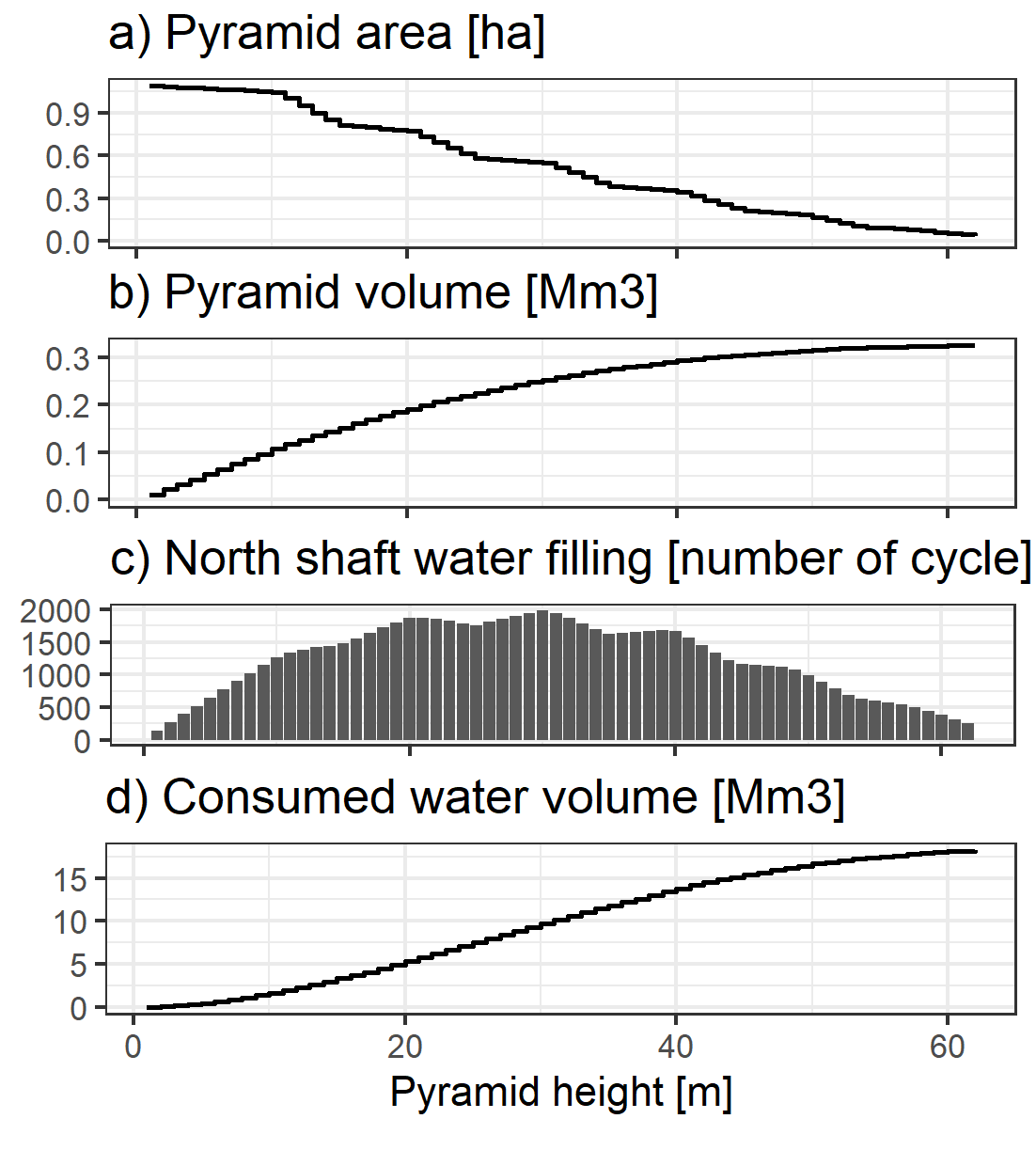


Fig. S8.

Pyramid building main quantities at various heights: a) pyramid structure area at a given height, b) cumulated volume below a given height, c) several cycles of filling and drainage of the north shaft to lift a given pyramid course, and d) cumulated water consumption to raise the pyramid until a given height.

Table S1. Parameters used in the hydraulic lift numerical model.

| **Parameters** | **Notation** | **Value** | **Units** |
| --- | --- | --- | --- |
|  | | | |
| ***Pyramid structure*** | | | |
| Pyramid total volume |  | 330 400 | m³ |
| Pyramid total height |  | 62.5 | m |
| Rock density | *ρ_r_* | 2600 | kg/m³ |
| Pyramid course thickness | *t* | 1 | m |
| Pyramid ground level | *Zq* | 55 | m ASL |
|  | | | |
| ***Central shaft*** | | | |
| Shaft side length | *c* | 7 | m |
| Shaft maximum water level | *Z_M_* | 45 | m ASL |
| Shaft minimum water level | *Z_m_* | 38 | m ASL |
| Shaft bottom level | *Z_b_* | 28 | m ASL |
| Box upper face height | *h_b_* | 5.4 | m |
|  | | | |
| ***Hydraulic lift*** | | | |
| Float side length | *a* | 6.5 | m |
| Float height | *h* | 3 | m |
| Float density | *ρ_f_* | 560 | kg/m³ |
| Water density | *ρ* | 1000 | kg/m³ |
| Extension weight | *w_e_* | 11 110 | kg |

**REFERENCES**

1. Friedt JM. Reconstruction de structures tridimensionnelles par photographies: le logiciel MicMac. OpenSilicium. 2014.

2. Reader C. An Early Dynastic Ritual Landscape at North Saqqara: An Inheritance from Abydos? The Journal of Egyptian Archaeology. 2017;103(1):71-87.

3. Farr TG, Elachi C, Hartl P, Chowdhury K. Microwave penetration and attenuation in desert soil: A field experiment with the Shuttle Imaging Radar. IEEE transactions on geoscience and remote sensing. 1986(4):590-4.

4. Elachi C, Roth LE, Schaber GG. Spaceborne radar subsurface imaging in hyperarid regions. IEEE Transactions on Geoscience and Remote Sensing. 1984(4):383-8.

5. Paillou P. Mapping palaeohydrography in deserts: contribution from space-borne imaging radar. Water. 2017;9(3):194.

6. Saad ZY. Royal excavations at Saqqarah and Helwan. Le Caire; 1947.

7. Kuraszkiewicz KO. Long Live the Step Pyramid! Heritage. 2022;5(3):2615-27.

8. Swelim N. Reminders and remarks on the royal substructure of the third dynasty. Mitteilungen des Deutschen Archäologischen Instituts, Abteilung Kairo. 2014;70:431-44.

9. Herbich T, Jagodziñski A. Geophysical investigation of the Dry Moat of the Netjerykhet complex in Saqqara. Man, millennia, environment: studies in honour of Romuald Schild. 2008:273-8.

10. Noc E. Analyse spatiale à Saqqâra des origines à la fin de l'Ancien Empire: les exemples des complexes funéraires de Netjerikhet et de Sekhemkhet: Montpellier 3; 2015.

11. Klemm D, Klemm R. Stones of the pyramids: German Archeological Institute, Cairo Department; 2010.

12. Lauer J-P. Histoire monumentale des pyramides d'Egypte. T. 1. Les pyramides à degrés (III. dynastie). 1-2. Bibliothèque d'étude. 1962;39:74.

13. Lauer J-P. La Pyramide à degrés - TOME 1 et 2. In: Orientale IFAO, editor. La Pyramide à degrés. Le Caire1936.

14. Monnier F. L'ère des géants: une description détaillée des grandes pyramides d'Egypte: Éditions De Boccard; 2017.

15. Stadelmann R. Die ägyptischen Pyramiden. Vom Ziegelbau zum Weltwunder. 1985;19922:147.

16. Stadelmann R. Builders of the Pyramids. Civilizations of the Ancient Near East. 1995;2:719-34.

17. Deslandes B. Travaux récents menés dans la pyramide à degrés de Saqqarah. Comptes-rendus des séances de l'année - Académie des inscriptions et belles-lettres. 2007;151(4):1475-82.

18. Deslandes B. Travaux récents dans la pyramide de Djeser (Nécropole de Saqqarah, Egypte). 2012.

19. Mays L, Antoniou GP, Angelakis AN. History of water cisterns: legacies and lessons. Water. 2013;5(4):1916-40.

20. Dixon DM. Timber in ancient Egypt. The Commonwealth Forestry Review. 1974;53(3 (157):205-9.

21. Jenkins N. The Boat Beneath the Pyramid Holt RaW, editor. 383 Madison Avenue

New York, New York1980.
